# Age and sex differences in the efficacy of early invasive strategy for non-ST-elevation acute coronary syndrome: A comparative analysis in stable patients

**DOI:** 10.1016/j.ajpc.2025.100984

**Published:** 2025-03-29

**Authors:** Edina Cenko, Maria Bergami, Jinsung Yoon, Giuseppe Vadalà, Sasko Kedev, Jorgo Kostov, Marija Vavlukis, Elif Vraynko, Davor Miličić, Zorana Vasiljevic, Marija Zdravkovic, Alfredo R. Galassi, Olivia Manfrini, Raffaele Bugiardini

**Affiliations:** a Laboratory of Epidemiological and Clinical Cardiology. Department of Medical and Surgical Sciences, University of Bologna, Bologna, Italy; b Google Cloud AI, Sunnyvale, CA, USA; c Division of Cardiology, University Hospital Paolo Giaccone, Palermo, Italy; d University Clinic for Cardiology, Skopje, Republic of North Macedonia; e Faculty of Medicine, Ss. Cyril and Methodius University in Skopje, 1000 Skopje, Republic of North Macedonia; f Department for Cardiovascular Diseases, University Hospital Center Zagreb, University of Zagreb, Zagreb, Croatia; g Medical Faculty, University of Belgrade, Belgrade, Serbia; h Faculty of Medicine University of Belgrade, Clinical Hospital Center Bezanijska kosa Belgrade Serbia; i Department of Health Promotion, Mother and Child Care, Internal Medicine and Medical Specialties (ProMISE), University of Palermo, Palermo, Italy; j IRCCS Azienda Ospedaliero-Universitaria di Bologna, Sant'Orsola Hospital, Bologna, Italy

**Keywords:** NSTE-ACS, Women, Revascularization, Risk stratification, Mortality

## Abstract

**Objective:**

Previous works have struggled to clearly define sex-specific outcomes based on initial management in NSTE-ACS patients. We examined if early revascularization (<24 h) versus conservative strategy impacts differently based on sex and age in stable NSTE-ACS patients upon hospital admission.

**Methods:**

We identified 8905 patients with diagnosis of non‐ST elevation acute coronary syndromes (NSTE-ACS) in the ISACS-TC database. Patients with cardiac arrest, hemodynamic instability, and serious ventricular arrhythmias were excluded. The final cohort consisted of 7589 patients. The characteristics between groups were adjusted using inverse probability of treatment weighting models. Primary outcome measure was all-cause 30-day mortality. Risk ratios (RRs) with their 95 % CIs were employed.

**Results:**

Of the 7589 NSTE-ACS patients identified, 2450 (32.3 %) were women. The data show a notable reduction in mortality for the older women (aged 65 years and older) undergoing early invasive strategy compared to those receiving an initial conservative (3.0 % versus 5.1 %; RR: 0.57; 95 % CI: 0.32 – 0.99) Conversely, younger women did not exhibit a significant association between early invasive strategy and mortality reduction (2.0 % versus 0.9 %; RR: 2.27; 95 % CI: 0.73 – 7.04). For men, age stratification did not markedly alter the observed benefits of an early invasive strategy over a conservative approach in the overall population, with reduced death rates in both older (3.1 % versus 5.7 %; RR: 0.52; 95 % CI: 0.34 – 0.80) and younger age groups (0.8 % versus 1.7 %; RR: 0.46; 95 % CI: 0.22 – 0.94). These age and sex-specific mortality patterns did not significantly change within subgroups stratified by the presence of NSTEMI, or a GRACE risk score>140.

**Conclusion:**

Early coronary revascularization is associated with improved 30-day survival in older men and women and younger men who present to hospital in stable conditions after NSTE-ACS. It does not confer a survival advantage in young women. Further studies are needed to more accurately risk-stratify young women to guide treatment strategies.

**Registration:**

ClinicalTrials.gov: NCT01218776

## Highlights

• Age and sex have a significant impact on the effectiveness of early invasive strategy in NSTE-ACS.
• An early invasive strategy is associated with a decrease in 30-day mortality among older women (65 years and older); a benefit not observed in younger women.
• Men benefit from an early invasive strategy irrespective of their age.
• These findings emphasize the need for personalized treatment plans considering age and sex for NSTE-ACS patients.

## Graphical abstract

**Figure:**
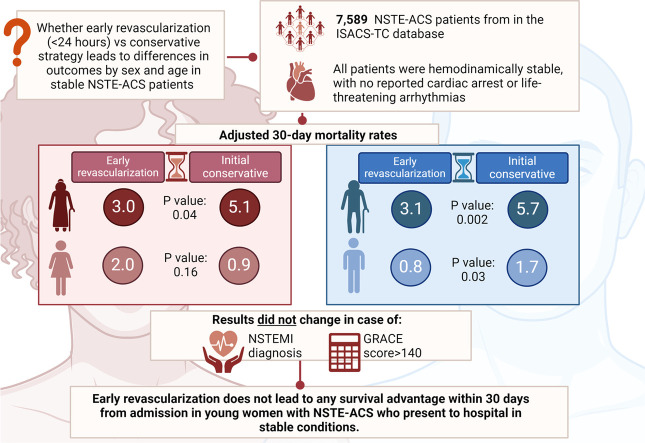

## 1 Introduction

Current guidelines for managing acute coronary syndromes (ACS) recommend an early invasive strategy within 24 h for patients diagnosed with non-ST-segment elevation myocardial infarction (NSTEMI), irrespective of sex [1]. Yet, this broad recommendation may not fully capture the subtle differences in clinical presentation, risk factors, and outcomes across sexes.

Remarkably, cardiovascular diseases typically present at a later age in women than in men, with the mean age at presentation being 62 years for men and 68 years for women, where age itself significantly predicts mortality [2,3]. Moreover, women with ACS are more prone to comorbidities like diabetes mellitus, hypertension, hypercholesterolemia, peripheral vascular disease, and often present with acute heart failure more frequently than their male counterparts [4,5]. Comorbid conditions increase the risk of disability and mortality over and above the risk from individual diseases [6]. There is, therefore, considerable doubt among physicians on how to handle these patients because of uncertainty about risk vs benefit in any treatment strategy.

Further complicating matters is the smaller size of coronary vessels in women compared with men, which challenges the effectiveness and safety of percutaneous and surgical revascularization [[7], [8], [9]]. This anatomical disparity potentially increases the risk of bleeding during invasive procedures [10]. Finally, post-hoc analyses of randomized trials have struggled to conclusively address sex-specific outcomes, leading to conflicting findings in meta-analyses [11,12]. These findings underscore the existing uncertainty around the risk-benefit balance of early invasive strategies in women with NSTEMI and non-ST-segment elevation acute coronary syndromes (NSTE-ACS).

Recognizing instances where urgent revascularization is clearly necessary such as in the case of life-threatening arrhythmias, cardiac arrest, cardiogenic shock, and severe heart failure regardless of the patient's sex or age, our study focused on a cohort of “stable” NSTE-ACS patients. In these patients, we aimed to explore the potential benefits and risks of an early invasive strategy compared with an initial conservative management approach, dissecting outcomes by sex. Furthermore, we assessed how the combination of age and sex might influence risk stratification, providing insights beyond the prognostic capabilities of existing risk scores along with the use of a clinical diagnosis of NSTEMI.

## 2 Methods

### 2.1 Study design and setting

The International Survey of Acute Coronary Syndromes (ISASC-TC; clinicaltrials.gov: NCT01218776) is a large, prospective, multicentre cohort study. Details of the study design, sampling, and recruitment have been previously published [[13], [14], [15], [16], [17], [18]]. Adhesion to work backward to define the 'nice to know' data elements on revascularization complications necessary to implement the present analysis plan was given by 8 collaborating centres from 7 European countries: Bosnia and Herzegovina, Croatia, Italy, Macedonia, Montenegro, Romania, and Serbia. All these centres were tertiary health care services providing PCI and cardiac surgery. This study complies with the Declaration of Helsinki [19]. The data-coordinating centre has been established at the University of Bologna. The local research ethics committee from each hospital approved the study. Because patient information was collected anonymously, institutional review boards waived the need for individual-informed consent. All data were transferred to the Department of Electrical and Computer Engineering, University of California, Los Angeles, where final statistical analyses were done.

### 2.2 Study population

The designated physician collected the registry data at the time of clinical assessment. All eligible patients must have presented to the hospital with chest pain occurring no >24 h prior to admission. In addition to chest pain, patients must have documented ST-segment depression on the ECG and/or evidence of myocardial necrosis (troponin concentration >99th centile using sex-specific upper reference limit on presentation or subsequent testing) [20]. An early invasive strategy was defined as coronary angiography with or without revascularization, either percutaneous coronary intervention (PCI) or coronary artery bypass graft (CABG), with procedure time being within 24 h of admission. The remaining patients were defined as an initial conservative strategy group. This definition of an early invasive strategy in NSTE‐ACS has previously been used for other large observational studies [[18], [21], [22], [23]]. The selection of the mode of revascularization (PCI or CABG) was based on patients' characteristics and preferences. Information regarding presence of diabetes was extracted from medical charts and information supplied by the patient. All patients categorized as having diabetes were on current antidiabetic medications. Information on diabetes was collected blinded to the outcomes.

### 2.3 Eligibility criteria

To meet eligibility criteria, patients had to be admitted in clinically stable conditions. We, therefore, applied the following exclusion criteria: life-threatening arrhythmias or cardiac arrest after presentation, cardiogenic shock (Killip class 4), acute severe heart failure (Killip class 3). These criteria would have suggested immediate urgent revascularization being the favoured therapeutic approach as opposed to initial conservative strategy [24,25]. To avoid immortal time bias - as patients who were selected for the study would have to survive enough to have the procedure - a landmark analysis was used. We defined the landmark time as 24 h from time of hospitalization. The analysis evaluated patient outcomes from the landmark time through to the end of the follow-up period, censored at 30 days from date of hospitalization.

### 2.4 Patient selection on the intention-to-treat principle and efficacy of revascularization

There were patients undergoing angiography within 24 h who did not receive revascularization. This suggests that no significant lesion was found, or that revascularization was deemed unnecessary or inappropriate. Including these patients in the early invasive strategy group would be a logical decision, based on the intention-to-treat principle. However, some considerations should be done. The primary benefit of revascularization in NSTE-ACS is typically observed in patients with significant coronary lesions. Including patients who underwent angiography within 24 h, but did not receive revascularization could potentially dilute the potential benefits. This is because their risk profile and outcomes could be substantially different from those who required revascularization. This concern addresses a key principle in clinical research: ensuring that the study population accurately reflects the intervention being evaluated. To circumvent this issue, we conducted the primary analyses both with and without these patients. This approach allows for a clearer interpretation of the data enabling us to compare how the inclusion of these patients affects the efficacy of the early invasive strategy.

### 2.5 Outcomes

Primary outcome measure of the study was all-cause 30-day mortality. The 30-day window for mortality was selected to enrich the data over that acquired during the index hospitalization while mitigating survivor bias. Other outcomes of interest were major bleeding and PCI complications. Major bleeding was defined as a decrease in blood hemoglobin level of at least 5 g/dL, the occurrence of intracranial hemorrhage or cardiac tamponade, fatal bleeding, or any combination of these events [26]. PCI complications that may have had significant impact on patient survival were rare. As such, they were combined in a single variable including: no-reflow (Thrombolysis in Myocardial Infarction [TIMI] 0–2 grading system) [27], coronary perforation or dissection, acute coronary thrombosis, coronary artery side branch loss, distal embolization, and elevated troponin post PCI intervention (levels of hs-cTn were determined within 6 h before the index procedure and in at least one sample after 4 to 6 h after PCI after 4 to 6 h after PCI) [20]. We did not include recurrence of ischemic events in our outcome measures as they are often driven by “symptoms of ischemia” but what this entails is uncertain, and, therefore, is a soft endpoint at risk of bias [28].

### 2.6 Concomitant therapies and definitions

We also noted the type of evidence-based medications given on hospital admission and during hospitalization until discharge. Medical therapy on admission included aspirin and P2Y_12_ inhibitors. Other standard treatments were given during hospitalization including angiotensin-converting enzyme inhibitors (ACE-inhibitors), angiotensin receptor blockers (ARB), beta blockers and statins. However, information on timing of in-hospital medications’ initiation was not systematically available in the database. As such, analyses on their effects on outcomes were not evaluated due to the possible persistence of immortal time bias. Smoking habits were self-reported. Hypertension and hypercholesterolemia were assessed by documentation of medical history prior to admission in the database (**Supplemental Methods**). The Global Registry of Acute Coronary Events (GRACE) risk score was calculated for each patient [29]. All patients with a glomerular filtration rate<60 mL/min/1.73 m^2^ for 3 months were defined as having chronic kidney disease [30]. Based on the coronary arteriographic findings, multivessel disease was defined as at least 2 main branches of the epicardial coronary artery with ≥70 stenotic lesions or ≥50 stenosis in the left main coronary artery.

### 2.7 Statistical analysis

We compared the baseline characteristics and clinical outcomes of patients who received an initial conservative strategy with those who received an early invasive strategy. Analyses were stratified by age (<65 years or ≥65 years) and sex. Other exploratory analyses included the criteria indicative of increased risk: NSTEMI and GRACE risk score of >140. Baseline characteristics were reported as number (percentages) for categorical variables and mean ± standard deviation for continuous variables. Statistical testing was performed with the use of the Pearson's chi-square test (χ2) for categorical variables and the two-sample *t*-test for continuous variables. A two-sided *P*-value of <0.05 was considered statistically significant. Each patient record detailed 23 clinical features and 8 medications (Table 1, Table 2). We used inverse probability of treatment weighting (IPTW) based on the propensity score for confounding adjustment (**Supplemental Methods**). To reduce the imbalance of potential confounding factors between the two treatment strategies, we compiled a set of baseline covariates as listed in Table 2. Variables included in the models were demographic, cardiovascular risk factors, comorbidities (history of ischemic heart disease, cardiovascular disease, and other comorbidities, namely chronic kidney disease) and clinical features on hospital presentation. We had complete data on sex and 30-day mortality. Among the variables included in the IPTW models, missingness was not considerable (<30) [31] (**Supplemental Table 1**). We used Multiple Imputation with Chained Equations (MICE) as imputation method to treat missing data [32] (**Supplemental Methods**). We reported the coefficient estimates, clustered adjust standard errors, T statistics, and corresponding p-values in **Supplemental Table 2**. Standardized differences after weighting were calculated to ensure balanced treatment groups with respect to baseline characteristics. Groups were considered balanced when the standardized difference was 10 % (**Supplemental Methods**). Risk ratios (ORs) and odds ratios (ORs) with their 95 confidence intervals (CIs) were employed (**Supplemental Methods**). Comparisons of outcomes between groups were made by two-sided *P*-value of <0.05 (**Supplemental Methods**). All statistical analyses were performed using R, version 4.2.1 (R Foundation for Statistical Computing, Vienna, Austria).

**Table 1:** Baseline characteristics.

>Characteristics	>Women			>Men		
	>Early invasive strategy (*N* >= >995)	>Initial conservative treatment (*N* >= >1455)	>Standardized mean difference	>Early invasive strategy (*N* >= >2518)	>Initial conservative treatment (*N* >= >2621)	>Standardized mean difference
Age, years, mean (SD)	66.2 (10.6)	68.1 (11.7)	−0.17	62.0 (10.9)	64.1 (12.0)	−0.18
**Cardiovascular risk factors**						
Diabetes, n ( %)	352 (35.4)	544 (37.4)	−0.04	64 (26.4)	783 (29.9)	−0.08
Hypercholesterolemia, n ( %)	536 (53.9)	716 (49.2)	0.09	1237 (49.1)	1190 (45.4)	0.07
Hypertension, n ( %)	831 (83.5)	1203 (82.7)	0.02	1848 (73.4)	2018 (77.0)	−0.08
Current smokers, n ( %)	279 (28.0)	282 (19.4)	0.20	1028 (40.8)	897 (34.2)	0.14
Family history of CAD, n ( %)	353 (35.5)	528 (36.3)	−0.02	909 (36.1)	857 (32.7)	0.07
**Cardiovascular history**						
Chronic coronary syndrome, n ( %)	269 (27.0)	532 (36.6)	−0.21	665 (26.4)	835 (31.9)	−0.12
Prior myocardial infarction, n ( %)	187 (18.0)	322 (22.1)	−0.08	578 (23.0)	675 (25.8)	−0.06
Prior CABG, n ( %)	20 (2.0)	54 (3.7)	−0.10	77 (3.1)	175 (6.7)	−0.17
Prior PCI, n ( %)	155 (15.6)	155 (10.7)	0.15	480 (19.1)	397 (15.1)	0.10
Peripheral artery disease, n ( %)	46 (4.6)	42 (2.9)	0.09	85 (3.4)	108 (4.1)	−0.04
Prior heart failure, n ( %)	63 (6.3)	115 (7.9)	−0.06	96 (3.8)	188 (7.2)	−0.15
Prior stroke or TIA, n ( %)	50 (5.0)	87 (6.0)	−0.04	81 (3.2)	155 (5.9)	−0.13
**Comorbidities**						
Chronic kidney disease, n ( %)	98 (9.8)	144 (9.9)	−0.002	176 (7.0)	256 (9.8)	−0.1
HR, bpm, mean (SD), n ( %)	80.8 (18.2)	85.2 (21.4)	−0.22	79.4 (18.3)	83.8 (20.8)	−0.23
SBP, mmHg, mean (SD), n ( %)	145.9 (25.9)	142.9 (26.5)	0.11	143.6 (25.5)	142.3 (25.7)	0.0503
**ACS type**						
NSTEMI, n ( %)	826 (83.0)	1072 (73.7)	0.23	2050 (81.4)	1979 (75.5)	0.14
Unstable angina n ( %)	169 (17.0)	380 (26.1)	−0.22	468 (18.6)	630 (4.0)	−0.13
**Prior medications**						
Antiplatelet medications, n ( %)	506 (50.9)	720 (49.5)	0.03	1122 (44.6)	1200 (45.8)	−0.02
ACE-inhibitors or ARBs, n ( %)	552 (55.5)	884 (60.8)	−0.11	1272 (50.5)	1367 (52.2)	−0.03
Beta blockers, n ( %)	490 (49.2)	734 (50.4)	−0.02	1071 (42.5)	1113 (42.5)	0.001
Statins, n ( %)	422 (42.4)	529 (36.4)	0.12	890 (35.3)	901 (34.4)	0.02
**In-hospital medications**						
Antiplatelet medications, n ( %)	991 (99.6)	1404 (96.5)	0.23	2503 (99.4)	2565 (97.9)	0.13
Heparins, n ( %)	802 (80.6)	1199 (82.4)	−0.05	2040 (81.0)	2207 (84.2)	−0.08
Beta blockers, n ( %)	774 (77.8)	1195 (82.1)	−0.11	1887 (74.9)	2046 (78.1)	−0.07
Beta blockers within 24 h, n ( %)	439 (44.1)	547 (37.6)	0.13	1047 (41.6)	902 (34.4)	0.15
ACE-inhibitors/ARBs, n ( %)	805 (80.9)	1181 (81.2)	−0.01	1983 (78.8)	2056 (78.4)	0.01
ACE-inhibitors/ARBs within 24 h, n ( %)	492 (49.4)	573 (39.4)	0.20	1251 (49.7)	997 (38.0)	0.24
GP IIb/IIIa inhibitors, n ( %)	60 (6.0)	30 (2.1)	0.20	165 (6.6)	81 (3.1)	0.16
Statins, n ( %)	954 (95.9)	1310 (90.0)	0.23	2427 (96.4)	2422 (92.4)	0.17
PCI, n ( %)	982 (98.7)	341 (23.4)	2.42	2477 (98.4)	889 (33.9)	1.86
CABG, n ( %)	81 (8.1)	153 (10.5)	−0.08	200 (7.9)	254 (9.7)	−0.06
**Outcomes**						
30-day mortality, n ( %)	24 (2.4)	55 (3.8)	−0.08	(1.5)	108 (4.1)	−0.16
Major bleeding, n ( %)	12 (1.2)	25 (1.7)	−0.04	23 (0.9)	52 (2.0)	−0.09
PCI related complications, n ( %)	32 (3.2)	20 (1.4)	0.12	98 (3.9)	36 (1.4)	0.16

Data are presented as percentages ( %) or mean ±standard deviation, unless otherwise specified.

**Abbreviations:** ACE=angiotensin converting enzyme; ACS=acute coronary syndrome; ARBs=angiotensin receptor blockers; CABG=coronary artery bypass graft; CAD=coronary artery disease; GP=glycoprotein; HR=heart rate; NSTEMI=non-ST-segment elevation myocardial infarction; PCI=percutaneous coronary intervention; SBP=systolic blood pressure; TIA=transient ischemic attack.

**Table 2:** Clinical factors and 30-day mortality stratified by sex and treatment strategy (early invasive versus initial conservative): an inverse probability of treatment weighting analysis.

	>Women			>Men		
	>Early invasive strategy (*N* >= >995)	>Initial conservative treatment (*N* >= >1455)	>Standardized mean difference	>Early invasive strategy (*N* >= >2518)	>Initial conservative treatment (*N* >= >2621)	>Standardized mean difference
Age, years, mean (SD)	67.2 (10.6)	67.3 (11.8)	−0.01	62.9 (10.9)	62.9 (12.1)	−0.004
Diabetes, %	36.5	36.7	−0.01	27.7	27.8	−0.004
**Cardiovascular risk factors**						
Hypercholesterolemia, %	51.0	51.0	0.0005	47.0	47.0	−0.001
Hypertension, %	82.4	82.8	−0.01	75.2	75.1	0.002
Current smoking, %	23.0	23.0	−0.002	37.8	37.7	0.003
Family history of CAD, %	36.4	36.1	0.01	34.3	34.2	0.0004
**History of ischemic heart disease**						
Chronic coronary syndrome, %	32.3	32.6	−0.01	28.9	29.0	−0.004
Prior myocardial infarction, %	20.3	20.6	−0.01	24.1	24.3	−0.01
Prior CABG, %	3.3	3.1	0.01	4.8	4.9	−0.005
Prior PCI, %	12.6	12.6	0.001	17.2	17.1	0.002
**History of cardiovascular disease**						
Peripheral artery disease, %	3.4	3.4	−0.003	3.6	3.6	−0.003
Prior heart failure, %	7.2	7.2	0.003	5.5	5.5	−0.0001
Prior stroke or TIA, %	5.9	5.7	0.01	4.5	4.6	−0.003
**Other comorbidities**						
Chronic kidney disease, %	10.0	9.8	0.01	8.3	8.3	−0.001
**Clinical presentation on hospital admission**						
HR bpm, mean (SD)	83.6 (20.9)	83.4 (20.3)	0.01	81.8 (21.0)	81.7 (19.6)	0.01
SBP, mmHg, mean (SD)	143.7 (26.3)	143.9 (26.4)	−0.01	142.8 (26.0)	142.8 (25.2)	0.0001
**Outcomes**			***P-*value**			***P-*value**
30-day mortality, n ( %)	2.7	3.6	0.24	1.8	3.6	<0.001
Risk ratio (95 % CI)	0.76 (0.47 – 1.22)		0.25	0.49 (0.34 – 0.70)		<0.001

Data are weighted mean ± standard deviation or weighted rate, unless otherwise specified.

**Abbreviations:** CABG=coronary artery bypass graft; CAD=coronary artery disease; HR=heart rate; PCI=percutaneous coronary intervention; SBP=systolic blood pressure; TIA=transient ischemic attack.

## 3 Results

A total of 8905 with NSTE-ACS were enrolled from the ISACS-TC participating hospitals between October 2010 and July 2023. From this group, 258 patients were excluded because they had evidence of cardiogenic shock (Killip class 4) or acute heart failure (Killip class 3) on hospital presentation. In addition, 135 patients were excluded because they died (*n* = 67) or had life-threatening arrhythmias or cardiac arrest (*n* = 68) before the landmark time. Moreover, 923 patients were excluded as angiography was not followed by revascularization in the first 24 h (*n* = 565) or they had incomplete data on the timing of angiography (*n* = 358). The final cohort consisted of 7589 patients. Of these 2450 were women while 5139 were men (**Supplemental Figure 1**). Thereafter, we included the 565 patients who underwent angiography within 24 h but did not receive revascularization in sensitivity analyses. This inclusion may provide a more comprehensive view of the early invasive strategy outcomes.

### 3.1 Baseline characteristics of the overall study population

A total of 3513 patients (46.3 %) underwent an early invasive strategy during their admission. Of the 4076 patients who were treated with initial conservative strategy, 39.4 % underwent later revascularization during the initial hospitalization. The timing of revascularization of these patients is shown in **Supplemental Figure 2**. Cardiac procedures were carried out approximately two to three days after admission in both women and men.

### 3.2 Baseline characteristics stratified by sex and treatment strategy

A lower proportion of women than men (Table 1) underwent an early invasive strategy (40.6 % versus 49.0 %). Baseline differences between treatment strategy groups were similar among women and men. Compared with an initial conservative strategy, patients undergoing an early invasive strategy were significantly (standardized difference ≥10 %) younger, and more likely to be admitted to a cardiology service with a diagnosis of NSTEMI. Patients who received an early invasive strategy had lower unadjusted rates for 30-day mortality in men, but not in women.

### 3.3 Care patterns

Patients who underwent early invasive management were statistically more likely to receive antiplatelet agents on hospital admission compared with patients who underwent early conservative management either among women and men (Table 1).

### 3.4 Angiographic findings

**Supplemental Figure 3** demonstrates the significantly more widespread CAD among the male cohort, with as many as 39.2 % of these patients categorized as having multivessel CAD compared with 32.1 % of their female counterparts (*P*-value<0.001).

### 3.5 Outcomes stratified by sex after propensity weighting

Women and men in the early invasive and conservative groups were well balanced after IPTW with standardized difference <10 % for all covariates (Table 2). In the weighted sample, the 30-day mortality rate in women was not significantly lower with the early invasive strategy compared to the initial conservative strategy (2.7 % versus 3.6 %; RR: 0.76; 95 % CI: 0.47 – 1.22). Conversely, in men, adjustment for baseline characteristics highlighted a significant benefit of the early invasive management (1.8 % versus 3.6 %; RR: 0.49; 95 % CI: 0.34 – 0.70). This corresponds to a 51 % reduction in mortality risk for men, which was statistically significant, compared to a 24 % reduction for women, which was not statistically significant. However, the relative risks between the subgroups did not significantly differ from each other (*P*_interaction_ = 0.07) (**Supplemental Table 3**)."

### 3.6 Outcomes stratified by age and sex after propensity weighting

Stratifying patients by age revealed distinct differences in risk between women and men. Among older women (aged 65 years and older), early invasive strategy was associated with a significant reduction in mortality, showing an absolute death rate difference of 2.1 (3.0 % versus 5.1 %; RR: 0.57; 95 % CI: 0.32 – 0.99). Conversely, younger women did not exhibit a significant association between early invasive strategy and mortality reduction (2.0 % versus 0.9 %; RR: 2.27; 95 % CI: 0.73 –7.04; *P*_interaction_ = 0.02) (Table 3 **and Supplemental Table 4).** For men, age stratification did not markedly alter the observed benefits of an early invasive strategy over a conservative approach in the overall population, with reduced death rates in both older (3.1 % versus 5.7 %; RR: 0.52; 95 % CI: 0.34 – 0.80) and younger age groups (0.8 % versus 1.7 %; RR: 0.46; 95 % CI: 0.22 – 0.94; *P*_interaction_ = 0.39) (Table 4 **Supplemental Table 5).**

**Table 3:** Clinical factors and 30-day mortality stratified by age group and treatment strategy in women: an inverse probability of treatment weighting analysis.

	>Women < 65 years old			>Women ≥ 65 years old		
	>Early invasive strategy (*N* >= >423)	>Initial conservative treatment (*N* >= >537)	>Standardized mean difference	>Early invasive strategy (*N* >= >572)	>Initial conservative treatment (*N* >= >918)	>Standardized mean difference
Age, years, mean (SD)	55.8 (6.7)	55.9 (6.8)	−0.01	74.5 (6.1)	74.6 (6.6)	−0.01
Diabetes, %	27.9	27.7	0.004	42.8	42.8	−0.002
**Cardiovascular risk factors**						
Hypercholesterolemia, %	50.5	50.4	0.002	51.2	51.2	0.001
Hypertension, %	73.3	73.9	−0.01	88.4	88.7	−0.01
Current smoking, %	39.6	39.7	−0.002	12.0	12.2	−0.01
Family history of CAD, %	41.1	40.8	0.01	33.1	33.0	0.001
**History of ischemic heart disease**						
Chronic coronary syndrome, %	27.0	27.1	−0.002	35.9	36.2	−0.01
Prior myocardial infarction, %	17.1	17.8	−0.02	22.5	22.3	0.01
Prior CABG, %	1.4	1.4	−0.002	4.6	4.1	0.03
Prior PCI, %	10.8	10.8	−0.001	14.0	13.7	0.01
**History of cardiovascular disease**						
Peripheral artery disease, %	2.3	2.2	0.01	4.0	4.1	−0.01
Prior heart failure, %	3.7	3.9	−0.01	9.4	9.3	0.002
Prior stroke or TIA, %	3.5	3.6	−0.01	7.5	7.1	0.02
**Other comorbidities**						
Chronic kidney disease, %	4.0	3.9	0.004	13.9	13.7	0.01
**Clinical presentation on hospital admission**						
HR, bpm, mean (SD)	81.9 (19.1)	81.7 (17.6)	0.01	84.6 (21.9)	84.4 (21.8)	0.01
SBP, mmHg, mean (SD)	145.5 (26.8)	145.8 (25.8)	−0.01	142.6 (26.4)	142.8 (26.7)	−0.01
**Outcomes**			***P-*value**			***P-*value**
30-day mortality, n ( %)	2.0	0.9	0.16	3.0	5.1	0.04
Risk ratio (95 % CI)	2.27 (0.73 – 7.04)		0.09	0.57 (0.32 – 0.99)		0.04

Data are weighted mean ± standard deviation or weighted rate, unless otherwise specified.

**Abbreviations:** CABG=coronary artery bypass graft; CAD=coronary artery disease; HR=heart rate; PCI=percutaneous coronary intervention; SBP=systolic blood pressure; TIA=transient ischemic attack.

**Table 4:** Clinical factors and 30-day mortality stratified by age group and treatment strategy in men: an inverse probability of treatment weighting analysis.

	>Men <65 years old			>Men ≥ 65 years old		
	>Early invasive strategy (*N* >= >1454	Initial conservative treatment *N* = 1377)	>Standardized mean difference	>Early invasive strategy (*N* >= >1064)	>Initial conservative treatment (*N* >= >1244)	>Standardized mean difference
Age, years, mean, (SD)	54.6 (7.0)	54.6 (7.4)	0.01	73.3 (5.6)	73.3 (6.3)	−0.01
Diabetes, %	22.4	22.4	−0.001	34.7	34.7	0.001
**Cardiovascular risk factors**						
Hypercholesterolemia, %	47.9	47.9	0.00	46.2	45.8	0.01
Hypertension, %	68.5	68.4	0.003	84.1	84.0	0.004
Current smoking, %	50.3	50.2	0.001	22.2	22.2	0.001
Family history of CAD, %	38.7	38.7	0.00	28.2	28.4	−0.01
**History of ischemic heart disease**						
Chronic coronary syndrome, %	26.4	26.4	0.0002	32.2	32.4	−0.01
Prior myocardial infarction, %	21.9	21.9	−0.001	26.0	26.6	−0.01
Prior CABG, %	3.2	3.3	−0.003	6.8	6.9	−0.004
Prior PCI, %	16.7	16.6	0.003	17.7	17.8	−0.002
**History of cardiovascular disease**						
Peripheral artery disease, %	2.0	2.0	−0.001	5.3	5.5	−0.01
Prior heart failure, %	3.7	3.7	−0.002	7.9	7.7	0.004
Prior stroke or TIA, %	3.0	3.0	−0.004	6.6	6.4	0.01
**Other comorbidities**						
Chronic kidney disease, %	4.3	4.2	0.01	13.1	13.4	−0.01
**Clinical presentation on hospital admission**						
HR, bpm, mean (SD)	81.5 (20.1)	81.3 (18.9)	0.01	82.1 (21.6)	82.1 (20.6)	0.001
SBP, mmHg, mean (SD)	143.3 (26.2)	143.2 (25.2)	0.001	142.3 (25.5)	142.2 (25.3)	0.003
**Outcomes**			***P-*value**			***P-*value**
30-day mortality, n ( %)	0.8	1.7	0.03	3.1	5.7	0.002
Risk ratio (95 % CI)	0.46 (0.22 – 0.94)		0.03	0.52 (0.34 – 0.80)		0.003

Data are weighted mean ± standard deviation or weighted rate, unless otherwise specified.

**Abbreviations:** CABG=coronary artery bypass graft; CAD=coronary artery disease; HR=heart rate; PCI=percutaneous coronary intervention; SBP=systolic blood pressure; TIA=transient ischemic attack.

### 3.7 Age and subgroup analyses based on high baseline risk

**Fig. 1:**
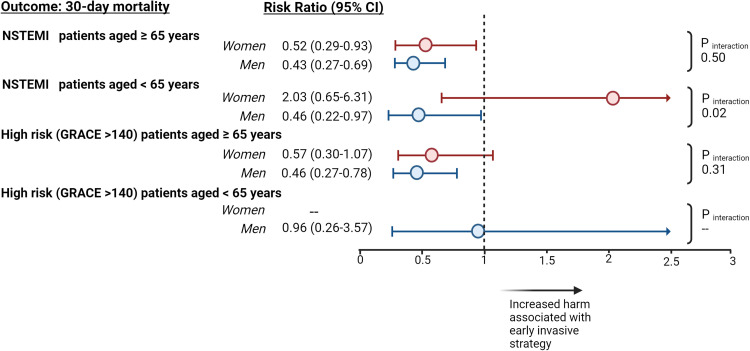
Inverse probability of treatment weighting analysis of the effect of early invasive versus initial conservative treatment strategy on 30-day mortality in patients with NSTEMI or GRACE risk score > 140, stratified by age and sex.

Abbreviations: NSTEMI, Non-ST elevation myocardial infarction. Figure created with Biorender

In comparison to our primary analysis, the sex-specific patterns of primary outcomes did not significantly change within subgroups stratified by age, the presence of NSTEMI, or a GRACE risk score above 140. For older patients with NSTEMI, both women and men experienced a remarkable decrease in mortality when subjected to an early invasive strategy with RRs of 0.52 (95 % CI: 0.29 – 0.93) for women and 0.43 (95 % CI: 0.27 – 0.69; *P*_interaction_ = 0.31) for men (Fig. 1 **and Supplemental Tables 6 and 7)**. Similarly, the advantages of an early invasive strategy over a conservative approach in reducing mortality remained consistent among younger men with NSTEMI (RR: 0.46; 95 % CI: 0.22 – 0.97). Yet, this correlation did not extend to younger women with NSTEMI, who did not exhibit significant mortality reduction associated with early invasive management (RR: 2.03; 95 % CI: 0.65 – 6.31; *P*_interaction_ = 0.02) (**Supplemental Tables 8 and 9)**. For older patients with a GRACE risk score higher than 140, the relative treatment effect of an early invasive versus initial conservative treatment on mortality was not different between sexes (RR for women: 0.57 [95 % CI: 0.30 - 1.07]; RR for men: 0.46 [95 % CI: 0.27 - 0.78]; *P*_interaction_ = 0.31) (**Supplemental Tables 10 and 11)**. The number of younger men and women with a GRACE risk score exceeding 140 was too low to definitively ascertain benefits within these specific subgroups (Fig. 1 **and Supplemental Table 12)**.

### 3.8 Subgroup analyses stratified by sex in low risk stable patients

Excluding patients with high-risk hemodynamic features, as done in our study, may have reduced the prognostic significance of the GRACE score. In patients with a GRACE score <140, the weighted prediction model still demonstrated a benefit of an early invasive strategy over a conservative approach in reducing mortality in men (RR: 0.51 [95 % CI: 0.29 – 0.88]), but this effect was not observed in women (RR: 1.15 [95 % CI: 0.48 – 2.72]) (**Supplemental Table 13 and 14**).

### 3.9 Safety outcomes

**Fig. 2:**
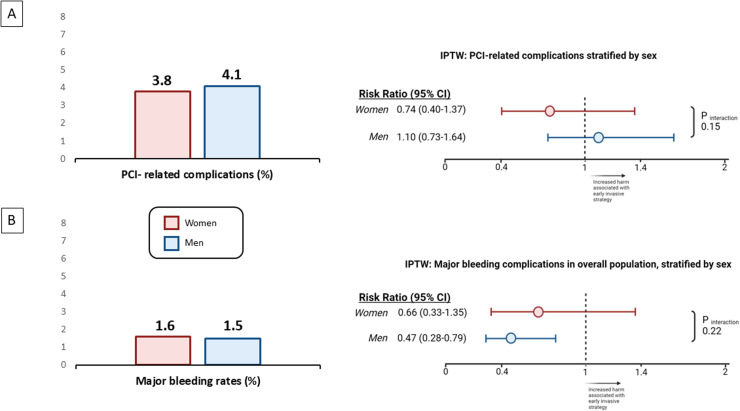
Inverse probability of treatment weighting analysis of the effect of early invasive versus initial conservative treatment strategy on safety outcomes. Panel A: On the left, weighted rates of PCI-related complications across sexes; On the right, effect of early invasive versus initial conservative treatment strategy on PCI-related complications, stratified by sex. Panel B: On the left, weighted rates of major bleeding complications across sexes; On the right, effect of early invasive versus initial conservative treatment strategy on major bleeding complications, stratified by sex.

Abbreviations: PCI, percutaneous coronary intervention. Figure created with Biorender

Among patients undergoing PCI as part of either an early invasive or conservative strategy, the incidence of PCI-related complications was found to be similar between women and men, with women experiencing a weighted rate of 3.8 % compared with 4.1 % in men (RR: 0.94; 95 % CI: 0.67 – 1.30) (Fig. 2A **and Supplemental Table 15**). Additionally, the complication rates between the early intervention and conservative strategy groups were comparable within both women (3.3 % versus 4.5 %, RR: 0.74; 95 % CI: 0.40 – 1.37) and men (4.0 % versus 3.7 %, RR: 1.10; 95 % CI: 0.73 – 1.64; *P*_interaction_ = 0.15), indicating no significant difference in the safety profile of PCI between the two strategies across sexes **(Supplemental Tables 16 and 17**). The overall weighted rate of major bleeding, as detailed in the Fig. 2B **and Supplemental Tables 18**, was comparable between women and men (1.6 % versus 1.5 %, RR: 1.08; 95 % CI: 0.73 – 1.59). Also, the relative treatment effect of an early invasive versus initial conservative was not different between sexes (RR for women: 0. 66 [95 % CI: 0.33 – 1.35]; RR for men: 0.47 [95 % CI: 0.28 – 0.79]; *P*_interaction_ = 0.22) **(Supplemental Tables 19 to 20)**.

### 3.10 Intention-to-Treat analysis

Our analysis adhered to the intention-to-treat principle, incorporating data from 565 patients who underwent early angiography but did not proceed to subsequent revascularization. This inclusion did not significantly alter the previously observed outcomes when these patients were excluded. Among older men, the early invasive strategy demonstrated a notable risk reduction, with an absolute mortality difference of 1.7 (RR: 0.68; 95 % CI: 0.47 – 0.99), highlighting significant benefits within this subgroup. A similar pattern emerged for younger men, showing an absolute mortality difference of 1.0 (RR: 0.47; 95 % CI: 0.24 – 0.89; *P*_interaction_ = 0.17) **(Supplemental Table 21 and 22)**. Conversely, the effect was age-specific among women. Older women showed a potential benefit (RR: 0.73 [95 % CI: 0.44 −1.20]), while younger women experienced a potential increased risk (RR: 2.50 [95 % CI: 0.83 – 7.55], *P*_interaction_ = 0.02). **(Supplemental Table 23 and 24).** This analysis highlights the variable effects of early invasive strategies across different sexes and age groups, even when including patients who underwent only diagnostic angiography without subsequent revascularization.

## 4 Discussion

This study offers new insights into the real-world management of patients with NSTE-ACS in a stable condition upon hospital admission. In this population, age and sex significantly influence the effectiveness of early invasive strategies. Older women (65 years and older) benefit from early invasive management with a notable reduction in mortality, whereas younger women do not show a similar benefit. In contrast, men of all ages benefit from early invasive strategies, underscoring the importance of personalized treatment plans that consider both age and sex in the management of NSTE-ACS.

### 4.1 Sex difference in the impact of an early invasive therapy in the population as a whole

Our findings on the differential impacts of an early invasive therapy in NSTE-ACS among women and men are in harmony with prior post-hoc analyses from key NSTE-ACS trials that did not implement risk stratification. Notably, the RITA-3 trial (The third Randomized Intervention Trial of unstable Angina) observed a paradoxical outcome where women experienced lower rates of death and myocardial infarction at one year with conservative management compared with invasive strategies (5.1 % versus 8.6 %) [33]. Similarly, the FRISC II trial (Fragmin and Fast Revascularization during Instability in Coronary artery disease) highlighted adverse outcomes for women undergoing invasive therapy at 12 months follow-up [34]. The TACTICS-TIMI 18 (Treat Angina with Aggrastat and determine Cost of Therapy with an Invasive or Conservative Strategy) trial further corroborated these sex disparities, showing a significant benefit from invasive management in men (OR: 0.64; 95 % CI, 0.47 – 0.88), but not in women (OR: 0.72; 95 % CI: 0.47 – 1.11) [35]. Our analysis aligns with these observations, revealing a pronounced benefit of invasive therapy in men (1.8 % versus 3.6 %; RR: 0.49; 95 % CI: 0.34 – 0.70) but a significantly diminished advantage in women (2.7 % versus 3.6 %; RR: 0.76; 95 % CI: 0.47 – 1.22). These findings collectively underline the complexity of managing NSTE-ACS among women and challenge the one-size-fits-all approach of current treatment guidelines. They advocate for a more individualized therapeutic strategy that considers both sex and detailed risk stratification to optimize patient outcomes.

### 4.2 Early coronary revascularization in a stable NSTE-ACS population

Previous studies that estimated the average treatment effect for NSTE-ACS often assumed a uniform response to treatment across a heterogeneous patient population, potentially overlooking the distinct clinical needs and outcomes of specific subgroups. It is in this context that the current study should be viewed. We examined 30-day survival among of 8920 NSTE-ACS presenting to hospital in “stable” clinical conditions. Often, previous work has encompassed patients experiencing recurrent ischemic episodes, significant ventricular arrhythmias, and hemodynamic instability who were not clearly in a stable phase of their disease requiring immediate invasive management within 2 h, without need of considering sex or detailed risk stratification. By excluding such patient groups from our analysis, we aimed to provide a clearer understanding of how treatment strategies differentially impact outcomes of women and men.

### 4.3 Age and heterogeneity of treatment effect

An important finding of our study was the predictive value of age. Remarkably, older patients (aged ≥65 years) benefitted substantially from an early invasive strategy as evidenced by a marked decrease in all-cause mortality rates, 2.1 % for women and 2.6 % for men, relative to those managed with an early conservative approach. For younger men, while the benefit of early invasive strategy persisted, it manifested in a less pronounced manner, with an absolute difference in mortality rate reduction of 0.9 %. In stark contrast, early coronary revascularization did not significantly reduce the risk of death among young women. These outcomes suggest a nuanced approach to managing stable patients with NSTE-ACS, where an early invasive strategy appears particularly beneficial for older patients, irrespective of their sex. For young women the urgency to undergo catheterization within the initial 24 h may not be critical, highlighting the importance of developing treatment strategies that are customized according to the patient's age and sex.

### 4.4 Patients categorized as high risk by guidelines

The efficacy of an early invasive strategy, particularly among the older, extended across various high-risk subgroups defined in the current ESC guidelines, including those with NSTEMI or a GRACE risk score greater than 140 [1]. Consistent with our primary findings, the benefit of an early invasive strategy over a conservative one was maintained for both older women and men, as well as younger men diagnosed with NSTEMI. Conversely, in the younger women cohort, the invasive strategy did not prove to be superior to the conservative approach. The differential impact of sex on treatment outcomes manifested more significantly among patients with GRACE risk score over 140. This was not unexpected, given that the GRACE risk score is heavily influenced by clinical variables like cardiac arrest, signs of heart failure, and hemodynamic instability, and women with NSTE-ACS exhibit signs of pulmonary congestion and worse Killip class more frequently than men [4,36]. In our study, we excluded patients presenting with these severe conditions, focusing instead on those in a stable phase of their disease. This exclusion inevitably led to sex-specific disparities in the predictive accuracy of the GRACE risk score. Within this framework, only older men with a GRACE score exceeding 140 showed a mortality reduction following the early invasive strategy, a benefit that was not mirrored in women [37]. Our findings, therefore, imply that the decision of when to perform early revascularization in patients with NSTE-ACS who are in stable condition at hospital admission should not rely solely on whether the patient has NSTEMI or a high GRACE risk score. A more personalized approach should be taken, considering additional factors. These factors might include the patient's overall health status and coexisting medical conditions which, in turn, can often be summarized by sex and age alongside clinical presentation.

### 4.5 Mechanisms of interplay between sex and age for CV outcomes

To our knowledge, there is no existing research on the interplay between sex, age, and the efficacy of early invasive versus conservative strategies in NSTE-ACS patients who present in "stable" conditions for direct comparison with our findings. While the specific mechanisms underlying the sex and treatment strategy interaction remain unclear, our data suggest that some hypotheses can be ruled out. Instances of rate of major bleeding and PCI-related complications of an early invasive therapy were infrequent, and there were no sex-based differences in strategy associated complications. The FRISC II investigators proposed that the diminished perceived benefits of interventions in women could be attributed to a lower prevalence of obstructive coronary artery disease at angiography, leading to fewer revascularizations [34]. In response, our study excluded participants with nonobstructive coronary lesions from the primary analysis, thus adhering to a crucial principle in clinical research: the precise definition of the study cohort to accurately reflect the intended target population for the intervention under investigation.

A potential reason for the age-based variation in the association of female sex with mortality after early coronary revascularization might stem from a greater prevalence of multiple NSTE-ACS causative factors in younger women, especially the combination of plaque rupture and microvascular dysfunction, the latter being more common in this age group [38]. In this regard, studies on PCI have shown that patients with pre-procedural impaired coronary flow reserve, a marker of microvascular dysfunction, are likely to sustain this impairment after the procedure, leading to worse outcomes [39]. Given the absence of data on coronary flow reserve, we can only speculate that ongoing myocardial ischemia, attributed to microvascular dysfunction, may neutralize the beneficial impact of early revascularization aimed at restoring coronary blood flow. This could potentially explain the observed absence of mortality reduction in young women.

### 4.6 Limitations

Our study should be interpreted in the context of several potential limitations. First, this analysis is not a randomized study. Although the propensity based IPTW helps to adjust for differences between groups, it does not control for unmeasured differences in clinical care. However, as a randomized trial cannot be carried out for every subgroup of patients, an observational database is helpful in providing hypothesis-generating data on the heterogeneity of treatment effects. Second, treatment algorithms might have changed over time between 2010 and 2023. Over a period of 13 years, advancements in the diagnostic and treatment modalities for NSTE-ACS have evolved significantly. The present results were obtained with limited use of second-generation ultrathin strut drug-eluting stents (DES). However, sex-based analyses of studies with second-generation DES in broad patient populations have suggested similar reductions in restenosis and repeat target vessel and target lesion revascularizations for both sexes [40]. Importantly, approximately 20 % of the included patients were biomarker negative according to conventional troponin assays, leading to their classification as unstable angina. It is conceivable that with the utilization of contemporary high-sensitivity troponin assays, the proportion of patients categorized as 'unstable angina' might actually be lower. This discrepancy may have attenuated the observable benefits of an early invasive strategy among those identified as NSTEMI. Nonetheless, the employment of IPTW to ensure a balanced distribution of unstable angina cases between the treatment arms serves to alleviate concerns regarding potential biases in the observed outcomes. Third, the present study did not define whether an early intervention should be a PCI or a CABG. This decision was at the discretion of the physicians. It is therefore not possible, on the basis of our data, to elaborate on the choice of revascularization procedure for women compared with men. Fourth, the limited duration of follow-up may obscure the possibility of later survival benefit. Finally, subgroup analyses can only be considered hypothesis generating as mentioned above.

### 4.7 Conclusions

Existing clinical guidelines do not currently incorporate age and sex alongside stable clinical presentation as factors for risk stratification in the context of selecting an early invasive versus conservative strategy. The current study demonstrates that the evidence contributing to the current treatment strategies is not as clearly defined as the guidelines might suggest.

Our analysis focuses on patients whose condition can safely be stabilised in the coronary care unit uncovering sex-specific variations in outcomes. Older patients experience the greatest benefit from early revascularization, irrespective of their sex. While younger male patients still benefit from an early invasive strategy, the impact is markedly less pronounced. In contrast, younger women do not show a similar benefit. If corroborated by other studies, these findings may have clinical implications for current management practices of patients who can be stabilized following NSTE-ACS. Additionally, the design of future trials may be structured to specifically account for these observed disparities.

## Source of funding

None.

## Author declaration

We wish to confirm that there are no known conflicts of interest associated with this publication and there has been no significant financial support for this work that could have influenced its outcome.

We confirm that the manuscript has been read and approved by all named authors and that there are no other persons who satisfied the criteria for authorship but are not listed. We further confirm that the order of authors listed in the manuscript has been approved by all of us.

We confirm that we have given due consideration to the protection of intellectual property associated with this work and that there are no impediments to publication, including the timing of publication, with respect to intellectual property.

In so doing we confirm that we have followed the regulations of our institutions concerning intellectual property.

We understand that the Corresponding Author is the sole contact for the Editorial process (including Editorial Manager and direct communications with the office).

He is responsible for communicating with the other authors about progress, submissions of revisions and final approval of proofs.

We confirm that we have provided a current, correct email address which is accessible by the Corresponding Author.

## CRediT authorship contribution statement

**Edina Cenko:** Writing – review & editing, Writing – original draft, Methodology, Investigation, Conceptualization. **Maria Bergami:** Writing – review & editing, Visualization. **Jinsung Yoon:** Methodology, Formal analysis. **Giuseppe Vadalà:** Writing – review & editing. **Sasko Kedev:** Writing – review & editing. **Jorgo Kostov:** Writing – review & editing. **Marija Vavlukis:** Writing – review & editing. **Elif Vraynko:** Writing – review & editing. **Davor Miličić:** Writing – review & editing. **Zorana Vasiljevic:** Writing – review & editing. **Marija Zdravkovic:** Writing – review & editing. **Alfredo R. Galassi:** Writing – review & editing. **Olivia Manfrini:** Writing – review & editing. **Raffaele Bugiardini:** Writing – review & editing, Writing – original draft, Validation, Methodology, Investigation, Conceptualization.

## Declaration of competing interest

The authors declare that they have no known competing financial interests or personal relationships that could have appeared to influence the work reported in this paper.

## Appendix Supplementary materials

Image, application 1
