# Supplementary material for: Age and sex differences in the efficacy of early invasive strategy for non-ST-elevation acute coronary syndrome: A comparative analysis in stable patients
Source: Am J Prev Cardiol. 2025 Mar 29;22:100984. doi: 10.1016/j.ajpc.2025.100984 (PMC12008549; doi:10.1016/j.ajpc.2025.100984)
Supplement: Supplementary file 1 [file mmc1.docx]

**SUPPLEMENTAL MATERIAL**

Table of Contents

[**SUPPLEMENTAL METHODS** 4](#_Toc188540678)

[**Risk factors definitions** 4](#_Toc188540679)

[**Multiple Imputation using Chained Equation (MICE) algorithm** 4](#_Toc188540680)

[**Inverse Propensity Score Weighting Analysis** 4](#_Toc188540681)

[**Computation of Relative Risk and its Confidence Interval** 5](#_Toc188540682)

[**Comparison of means and prevalences in the weighted sample** 6](#_Toc188540683)

[**Interaction tests** 7](#_Toc188540684)

[**SUPPLEMENTAL RESULTS** 8](#_Toc188540685)

[**Supplemental Figure 1**. Study flowchart. 8](#_Toc188540686)

[**Supplemental Figure 2**: Timing of revascularization in patients receiving initial conservative treatment strategy 9](#_Toc188540687)

[**Supplemental Figure 3**: Distribution of coronary artery disease characteristics 10](#_Toc188540688)

[**Supplemental Table 1.** Rates of missing values before Multiple Imputation using Chained Equation (MICE) algorithm 11](#_Toc188540689)

[**Supplemental Table 2.** General logistic regression and regression coefficients in the propensity score model in the overall NSTE-ACS population (early invasive vs. initial conservative strategy) 12](#_Toc188540690)

[**Supplemental Table 3**. Interaction test for comparing two estimated risk ratios (women vs. men): 30-day mortality for early invasive vs. initial conservative treatment 13](#_Toc188540691)

[**Supplemental Table 4**. Interaction test for comparing two estimated risk ratios in **women (age<65 years old vs. age ≥65 years old):** 30-day mortality for early invasive vs. initial conservative treatment 14](#_Toc188540692)

[**Supplemental Table 5**. Interaction test for comparing two estimated risk ratios in **men** (**age<65 years old vs. age ≥65 years old):** 30-day mortality for early invasive vs. initial conservative treatment 15](#_Toc188540693)

[**Supplemental Table 6.** Clinical factors and 30-day mortality stratified by sex and treatment strategy in **older patients (age ≥ 65 years old) presenting with NSTEMI:** inverse probability of treatment weighting 16](#_Toc188540694)

[**Supplemental Table 7.** Interaction test for comparing two estimated risk ratios (women vs. men) in **older patients (age ≥65 years** **old) presenting with NSTEMI**: 30-day mortality for early invasive vs. initial conservative treatment 18](#_Toc188540695)

[**Supplemental Table 8.** Clinical factors and 30-day mortality stratified by sex and treatment strategy in **younger patients** (**age<65 years old) presenting with NSTEMI:** inverse probability of treatment weighting 19](#_Toc188540696)

[**Supplemental Table 9**. Interaction test for comparing two estimated risk ratios (women vs. men) in **younger patients** (**age<65 years old) presenting with NSTEMI:**  30-day mortality for early invasive vs. initial conservative treatment 21](#_Toc188540697)

[**Supplemental Table 10.** Clinical factors and 30-day mortality stratified by sex and treatment strategy in **older patients** **(age≥65 years old)** with **GRACE score >140:** inverse probability of treatment weighting 22](#_Toc188540698)

[**Supplemental Table 11**. Interaction test for comparing two estimated risk ratios (women vs. men) in **older patients (age≥65 years old) with GRACE score >140**: 30-day for early invasive vs. initial conservative treatment 24](#_Toc188540699)

[**Supplemental Table 12.** Clinical factors and 30-day mortality stratified by sex and treatment strategy in **younger patients** (**age<65 years old)** **with GRACE score >140**: inverse probability of treatment weighting 25](#_Toc188540700)

[**Supplemental Table 13.** Clinical factors and 30-day mortality stratified by sex and treatment strategy in with **GRACE score<140:** inverse probability of treatment weighting 27](#_Toc188540701)

[**Supplemental Table 14**. Interaction test for comparing two estimated risk ratios (women vs. men) in **patients with GRACE score <140**: 30-day for early invasive vs. initial conservative treatment 29](#_Toc188540702)

[**Supplemental Table 15**. Clinical factors and PCI related complications stratified by sex in **patients undergoing PCI**: inverse probability of treatment weighting 30](#_Toc188540703)

[**Supplemental Table 16.** Clinical factors and PCI related complications stratified by sex and treatment strategy in patients **undergoing PCI**: inverse probability of treatment weighting 31](#_Toc188540704)

[**Supplemental Table 17.** Interaction test for comparing two estimated risk ratios (women vs. men) **in patients undergoing PCI:** PCI related complications for early invasive strategy vs. initial conservative treatment 33](#_Toc188540705)

[**Supplemental Table 18.** Clinical factors and **major bleeding complications** stratified by sex in **the overall population:** inverse probability of treatment weighting 34](#_Toc188540706)

[**Supplemental Table 19.** Clinical factors and **major bleeding complications** stratified by sex and treatment strategy in **the overall population:** inverse probability of treatment weighting 35](#_Toc188540707)

[**Supplemental Table 20**. Interaction test for comparing two estimated risk ratios (women vs. men): **major bleeding complications** in the overall population for early invasive vs. initial conservative treatment 37](#_Toc188540708)

[**Supplemental Table 21.** Clinical factors and 30-day mortality stratified by age group and treatment strategy in men: inverse probability of treatment weighting **(analysis incorporating patients undergoing coronary angiography without PCI**) 38](#_Toc188540709)

[**Supplemental Table 22**. Interaction test for comparing two estimated risk ratios in men (age<65 years old vs. age≥65 years old): 30-day mortality for early invasive strategy vs. initial conservative strategy (analysis incorporating patients undergoing coronary angiography without PCI) 40](#_Toc188540710)

[**Supplemental Table 23.** Clinical factors and 30-day mortality stratified by age group and treatment strategy in women: inverse probability of treatment weighting **(analysis incorporating patients undergoing coronary angiography without PCI**) 41](#_Toc188540711)

[**Supplemental Table 24.** Interaction test for comparing two estimated risk ratios in women (age<65 years old vs. age≥65 years old): 30-day mortality for early invasive strategy vs. initial conservative strategy (analysis incorporating patients undergoing coronary angiography without PCI) 43](#_Toc188540712)

[**REFERENCES** 44](#_Toc188540713)

# **SUPPLEMENTAL METHODS**

## **Risk factors definitions**

Smoking habits were self-reported. We defined current smokers as individuals who smoked 100 cigarettes in his or her lifetime and who smoked cigarettes, cigars, and cigarillos at the time of the index event. Everyday smokers or someday smokers were all included in this definition according to recommendations from the National Health Interview Survey^1^. Hypertension, hypercholesterolemia, and diabetes were assessed by designation of medical history prior to admission in the database. In addition, subjects taking medication because of arterial hypertension were classified to have arterial hypertension, even when blood pressure was controlled. Diabetes mellitus was also defined with regard to oral blood glucose lowering therapy or substitution of insulin. Positive family history of coronary artery disease (CAD) was defined as myocardial infarction, cardiac death, or need for coronary revascularization in a first-degree relative with early onset (under the age of 55 in men or 65 in women).

## **Multiple Imputation using Chained Equation (MICE) algorithm**

Multiple Imputation using Chained Equation (MICE) algorithm is an efficient and popular method to fill in missing data where each missing value on some records is replaced by a value obtained from related cases in the whole set of records. Thus, imputation for clinical features was conducted using the chained equations across other features^2^. More specifically, MICE algorithm sequentially imputes the missing values of clinical features based on both observed values and previously imputed values. This sequential imputation is conducted via chained equations.

We tried multiple imputations using the MICE algorithm for the initial analyses to address the uncertainty in the imputation process. More specifically, we generated multiple imputed datasets and check whether the conclusions are consistent across the different imputed datasets. If the conclusions are consistent across multiple imputed datasets, we use a single imputed dataset (by MICE algorithm) as the final dataset to report the results of statistical analyses in the paper.

## **Inverse Propensity Score Weighting Analysis**

We used Inverse Propensity Score Weighting (IPW) to balance the distribution of covariates between two patient groups. Note that we use Logistic Regression to estimate the propensity scores ({P}(Z=1 | x)). If *e* denotes the estimated propensity score (i.e. e=\hat{P}(Z=1 | x), where the patient x is included in patient group 1; then, 1-e = \hat{P}(Z=0 | x)), then the original sample is weighted by the following weights: Z/e+(1−Z)/ 1−e where Z represents the patient group. For instance, patients undergoing an early invasive strategy (Z=1) are assigned a weight equal to the reciprocal of the propensity score (1/e), while patients undergoing an initial conservative strategy (Z=0) are assigned a weight equal to the reciprocal of one minus the propensity score (1/1-e). The weighting procedure for each sample balances the covariate distributions between two patient groups^3^.

Inverse probability of treatment weighting method can potentially result in unstable and biased estimates if some of the weights are very high. To avoid excessive weights, we compared results with other methods for handling confounding. We included probability of treatment variables in a multivariable model. We also used XGBoost, a decision-tree-based ensemble machine learning algorithm, as an alternative multivariable model for estimating the probability of treatment. Conclusions from theses analyses were the same as our current results. Further, we created a threshold for weights to avoid the impacts of the outliers (we use 0.01 as threshold). Therefore, the inverse probability of treatment weighting analyses presented in the current analysis were quite stable.

## **Computation of Relative Risk and its Confidence Interval**

In a two-group cohort study, the risk ratio (RR, also called relative risk), is usually applied to compare risks of a health event between two independent binomial populations that differ by a demographic characteristic (i.e. sex, age) or by the level of exposure to a specific drug or risk factor. In such types of studies, data can be summarized in a confusion matrix as follows:

|  | **Risk of Designated Outcome** | |  |
| --- | --- | --- | --- |
|  | **Yes** | **No** | **Total** |
| **Exposed** | a | b | a+b (*H_1_*) |
| **Unexposed** | c | d | c+d (*H_0_*) |
| **Total** | a+c | b+d |  |

Where *H_1_* and *H_0_* correspond to the total number of exposed and unexposed patients, respectively, whereas *a* and *c* represent the number of exposed and unexposed patients at risk for the designated outcome, respectively.

RR is defined as the ratio between the risk of outcome in exposed patients (*H_1_*) and the risk of outcome in unexposed patients (*H_0,_*) which can be summarized as:

$$RR=\frac{\left( \frac{a}{H_{1}} \right)}{\left( \frac{c}{H_{0}} \right)}$$

When applying this equation to an IPTW balanced population, $\frac{a}{H_{1}}$ will be assigned a weight equal to the reciprocal of the propensity score ($\frac{1}{e}$) and $\frac{c}{H_{0}}$ will be weighted by the reciprocal of one minus the propensity score ($\frac{1}{(1-e)}$).

In order to compute the lower and upper (1-α) confidence limit RR_L_ for RR, we operate in the assumption of log normal distribution^4^. In particular, the variate $\log\frac{\left( \frac{a}{H_{1}} \right)}{\left( \frac{c}{H_{0}} \right)}$= $\log\frac{a}{H_{1}}- \log\frac{c}{H_{0}}$is approximately normally distributed with approximate mean log(RR) and estimated variance $\frac{1-\left( \frac{a}{H_{1}} \right)}{a}$ + $\frac{1-(\frac{c}{H_{0}})}{c}$ .

It follows that RR_L_ can be computed by solving the following equation:

$$\frac{\left[ log( \frac{\frac{a}{H_{1}}}{\frac{c}{H_{0}}})- \log({RR}_{L}) \right]}{\left[ \frac{1-\left( \frac{a}{H_{1}} \right)}{a} + \frac{1-(\frac{c}{H_{0}})}{c} \right]^{1/2}}=z_{1-\alpha}$$

Where $z_{1-\alpha}$, is the 100(1-α) percentage point of the N(O, 1) distribution

## **Comparison of means and prevalences in the weighted sample**

To evaluate the balance of the baseline covariate distributions between treatment and control groups, standardized difference (SD) is widely used in inverse probability of treatment weighting (IPTW) framework. For the baseline analysis, we use standard SD which is defined as follows: $\frac{m_{t}-m_{c}}{\sqrt{\frac{s_{t}^{2}+s_{c}^{2}}{2}}}$ for continuous variables and $\frac{m_{t}-m_{c}}{\sqrt{\frac{m_{t}(1-m_{t})+m_{c}(1-m_{c})}{2}}}$ for binary variable where $m_{t}, m_{c}$ are sample mean of the variables for treatment and control group, and $s_{t}^{2}, s_{c}^{2}$ are sample variance of the variables for treatment and control group, respectively. For IPTW analysis, we use weighted SD where $m_{t}, m_{c}$ are replaced to weighted sample mean of the variables for treatment and control group, and $s_{t}^{2}, s_{c}^{2}$ are replaced to weighted sample variance of the variables for treatment and control group, respectively. Weights are determined by the inverse probability of treatment received. In general, 0.1 is the reasonable threshold to determine whether two distributions are balanced (i.e., if SD > 0.1, the baseline covariate is imbalanced)^5^.

## **Interaction tests**

We tested **(Supplemental Table 3)** whether there is a significant interaction between sex (women versus men) and the treatment strategy of choice (early invasive vs. initial conservative treatment) in function of the outcome (30-day mortality)**.** We obtained the logs of the risk ratios and their confidence intervals (rows 2 and 4). As 95% confidence intervals were obtained as 1.96 standard errors (SE) on either side of the estimate, the SE of each log relative risk was obtained by dividing the width of its confidence interval by 2×1.96 (row 6). The estimated difference in log relative risks was *d*=E1- E2= 0.44 (row 7) and its standard error 0.31 (row 8). From these two values, we tested the interaction and estimated the ratio of the relative risks (with confidence interval). The test of interaction was the ratio of *d* to its standard error: z=1.78, which gave a *P* value=0.07 when we referred it to a table of the normal distribution (row 10). The estimated interaction effect was exp =1.44 (row 11). The confidence interval for this effect was -0.16-1.03 on the log scale (row 9). Transforming back to the relative risk scale, we got 0.85-2.82 (row 12).

# **SUPPLEMENTAL RESULTS**

## **Supplemental Figure 1**. Study flowchart.

ù
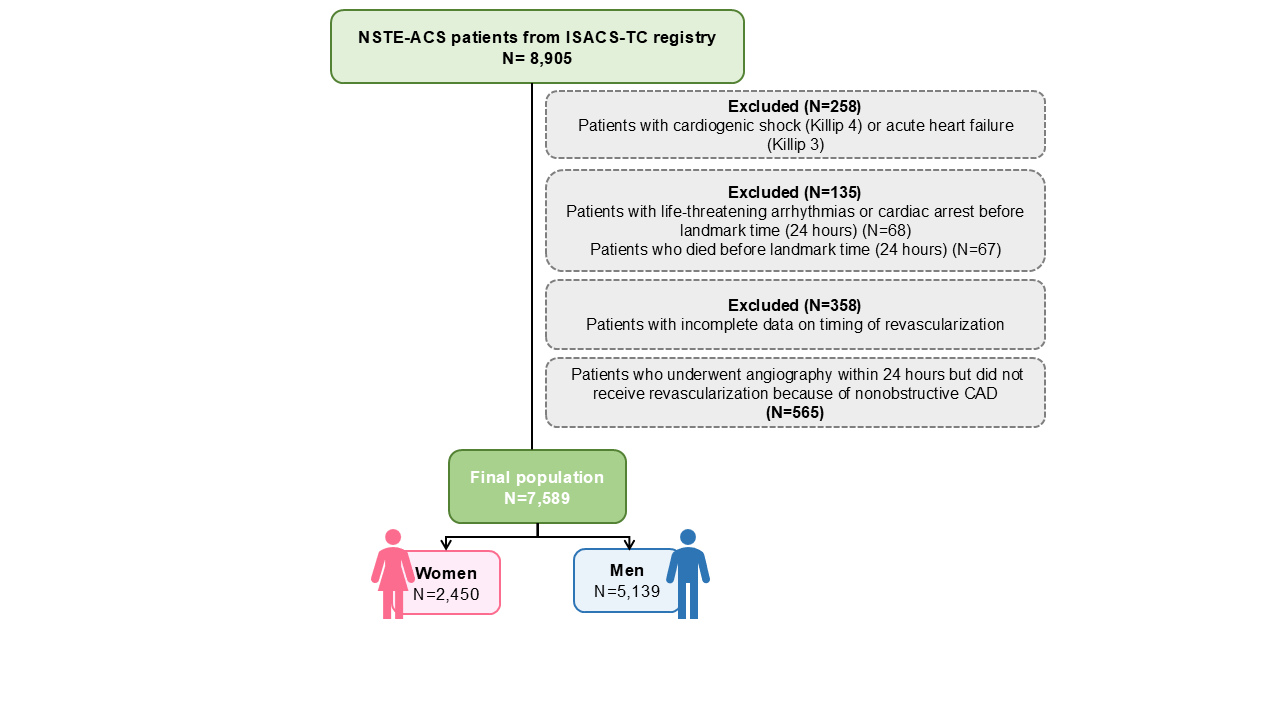


## **Supplemental Figure 2**: Timing of revascularization in patients receiving initial conservative treatment strategy


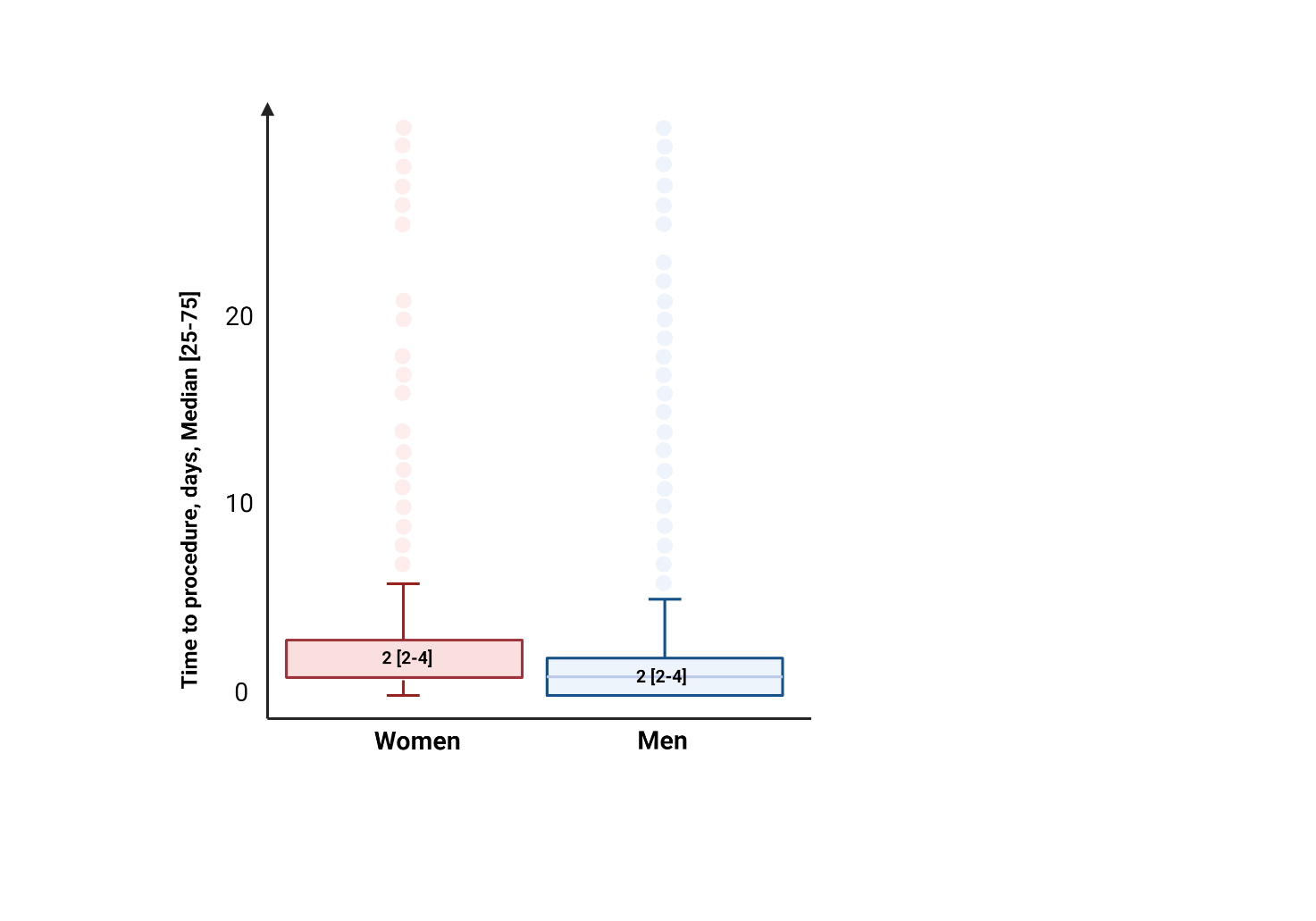


## **Supplemental Figure 3**: Distribution of coronary artery disease characteristics

**
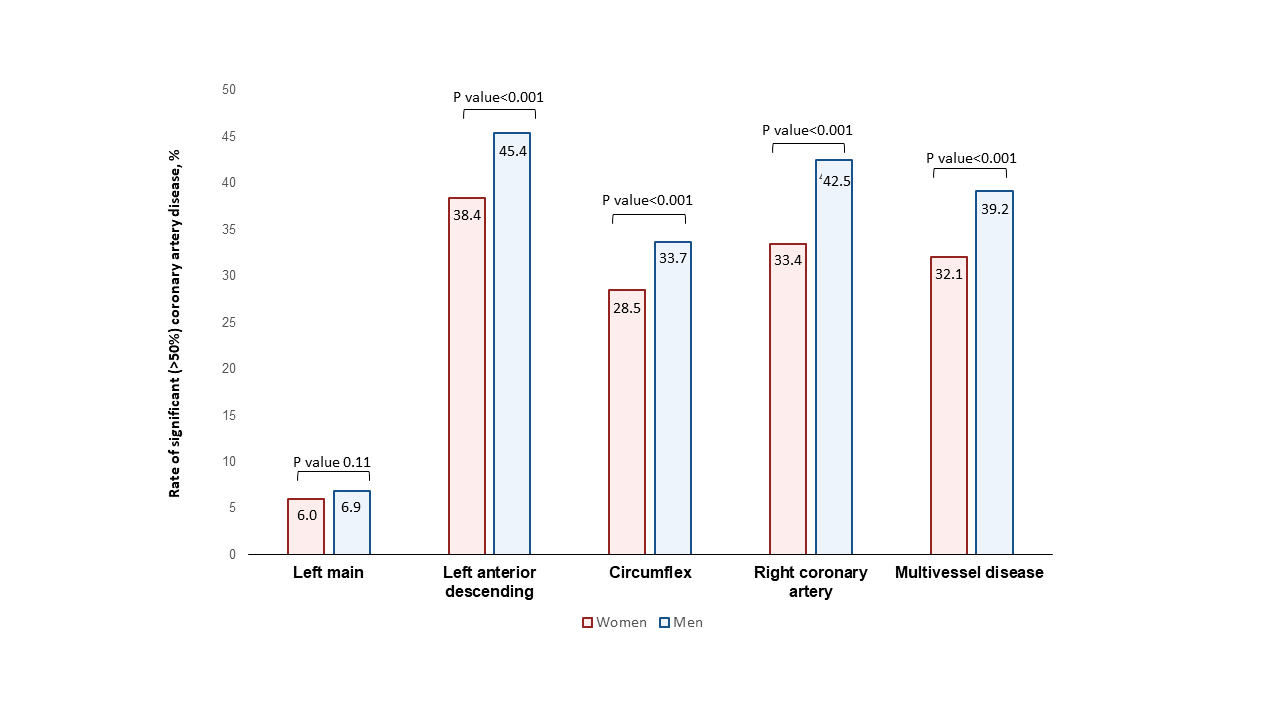
**

| **Supplemental Table 1.** Rates of missing values before Multiple Imputation using Chained Equation (MICE) algorithm | |
| --- | --- |
| **Variable name** | **Rate of missing values (%)** |
| Age | 1.4% |
| Female sex | 0.2% |
| **Cardiovascular risk factors** |  |
| Hypercholesterolemia | 7.1% |
| Hypertension | 1.0% |
| Current smoking | 3.8% |
| Family history of CAD | 10.4% |
| **History of ischemic heart disease** |  |
| Chronic coronary syndrome | 0.0% |
| Prior myocardial infarction | 0.0% |
| Prior CABG | 0.0% |
| Prior PCI | 0.0% |
| **History of cardiovascular disease** |  |
| Peripheral artery disease | 0.0% |
| Prior heart failure | 0.0% |
| Prior stroke or TIA | 0.0% |
| **Other comorbidities** |  |
| Chronic kidney disease | 27.4% |
| **Clinical presentation on hospital admission** |  |
| Heart rate | 18.1% |
| Systolic blood pressure | 18.4% |
| Data are presented as percentages (%)  **Abbreviations:** CABG=coronary artery bypass graft; CAD=coronary artery disease; PCI=percutaneous coronary intervention; TIA=Transient ischemic attack | |

| **Supplemental Table 2.** General logistic regression and regression coefficients in the propensity score model in the overall NSTE-ACS population (early invasive vs. initial conservative strategy) | | | | |
| --- | --- | --- | --- | --- |
|  | **β** | **SE** | **T statistics** | ***p* value** |
| **Constant term (α)** | 1.0065 | 0.2356 | 4.2719 | <0.0001 |
| Age | -0.0753 | 0.0233 | -3.2391 | 0.001 |
| Female sex | -0.2516 | 0.0522 | -4.8225 | <0.001 |
| Diabetes | -0.0231 | 0.0531 | -0.4360 | 0.66 |
| Hypercholesterolemia | 0.1834 | 0.0498 | 3.6817 | 0.0002 |
| Hypertension | -0.0735 | 0.0605 | -1.2156 | 0.22 |
| Current smoking | 0.1973 | 0.0540 | 3.6523 | 0.0003 |
| Family history of CAD | 0.0665 | 0.0527 | 1.2605 | 0.20 |
| Chronic coronary syndrome | -0.3756 | 0.0562 | -6.6808 | <0.001 |
| Prior myocardial infarction | -0.2527 | 0.0623 | -4.0556 | 0.0001 |
| Prior CABG | -0.6803 | 0.297 | -5.2455 | <0.001 |
| Prior PCI | 0.5704 | 0.0739 | 7.7220 | <0.001 |
| Peripheral artery disease | 0.1096 | 0.1282 | 0.8550 | 0.39 |
| Prior heart failure | -0.2418 | 0.1056 | -2.2896 | 0.02 |
| Prior stroke or TIA | -0.4548 | 0.1152 | -3.9497 | 0.0001 |
| Heart rate | -0.0108 | 0.0013 | -8.5285 | <0.001 |
| Systolic blood pressure | 0.0023 | 0.0009 | 2.4091 | 0.01 |
| Chronic kidney disease | -0.0357 | 0.0879 | -0.4062 | 0.68 |
| Optimized regression coefficient (ß) and constant term(α) for the logistic regression  **Abbreviations:** CABG=coronary artery bypass graft; CAD= coronary artery disease; NSTE-ACS=non-ST-segment elevation acute coronary syndrome PCI=percutaneous coronary intervention; TIA= Transient ischemic attack | | | | |

| **Supplemental Table 3**. Interaction test for comparing two estimated risk ratios (women vs. men): 30-day mortality for early invasive vs. initial conservative treatment | | | |
| --- | --- | --- | --- |
|  |  | **Group 1**  **[Women]**  **(n = 2450)** | **Group 2**  **[Men]**  **(n = 4727)** |
| **1** | **RR** | 0.76 | 0.49 |
| **2** | **log RR** | -0.27 | -0.71 |
| **3** | **95% CI for RR** | (0.47 – 1.22) | (0.34 – 0.70) |
| **4** | **95% CI for log RR** | -0.75-(0.20) | -1.07-(-0.36) |
| **5** | **Width of CI** | 0.95 | 0.72 |
| **6** | **SE (=width / (2*1.96))** | 0.24 | 0.18 |
|  | | | |
| **7** | **d (=**$\boldsymbol{E}_{\boldsymbol{1}}\boldsymbol{-}\boldsymbol{E}_{\boldsymbol{2}}$**)** | **0.44** | |
| **8** | **SE (d)** | **0.31** | |
| **9** | **CI (d)** | -0.16-1.03 | |
| **10** | **Test of Interaction** | 1.44 (***P* value=0.07**) | |
|  | | | |
| **11** | **RRR (=exp(d) )** | 1.55 | |
| **12** | **CI (RRR)** | 0.85-2.82 | |

| **Supplemental Table 4**. Interaction test for comparing two estimated risk ratios in **women (age<65 years old vs. age ≥65 years old):** 30-day mortality for early invasive vs. initial conservative treatment | | | |
| --- | --- | --- | --- |
|  |  | **Group 1**  **[Women age<65 years old]**  **(n = 1490)** | **Group 2**  **[Women age ≥65 years old]**  **(n = 960)** |
| **1** | **RR** | 2.27 | 0.57 |
| **2** | **log RR** | 0.82 | -0.56 |
| **3** | **95% CI for RR** | (0.73 – 7.04) | (0.32 – 0.99) |
| **4** | **95% CI for log RR** | -0.31-(1.95) | -1.13-(-0.01) |
| **5** | **Width of CI** | 2.27 | 1.13 |
| **6** | **SE (=width / (2*1.96))** | 0.58 | 0.29 |
|  | | | |
| **7** | **d (=**$\boldsymbol{E}_{\boldsymbol{1}}\boldsymbol{-}\boldsymbol{E}_{\boldsymbol{2}}$**)** | **1.38** | |
| **8** | **SE (d)** | **0.65** | |
| **9** | **CI (d)** | 2.65 | |
| **10** | **Test of Interaction** | 2.14 (***P* value=0.02**) | |
|  | | | |
| **11** | **RRR (=exp(d) )** | 3.98 | |
| **12** | **CI (RRR)** | 1.12-14.12 | |

| **Supplemental Table 5**. Interaction test for comparing two estimated risk ratios in **men** (**age<65 years old vs. age ≥65 years old):** 30-day mortality for early invasive vs. initial conservative treatment | | | |
| --- | --- | --- | --- |
|  |  | **Group 1**  **[Men age<65 years old]**  **(n =2831)** | **Group 2**  **[Men age ≥65 years old]**  **(n =2308)** |
| **1** | **RR** | 0.46 | 0.52 |
| **2** | **log RR** | -0.77 | -0.65 |
| **3** | **95% CI for RR** | (0.22 – 0.94) | (0.34 – 0.80) |
| **4** | **95% CI for log RR** | -1.51-(-0.06) | -1.07-(-0.22) |
| **5** | **Width of CI** | 1.45 | 0.86 |
| **6** | **SE (=width / (2*1.96))** | 0.37 | 0.22 |
|  | | | |
| **7** | **d (=**$\boldsymbol{E}_{\boldsymbol{1}}\boldsymbol{-}\boldsymbol{E}_{\boldsymbol{2}}$**)** | **-0.12** | |
| **8** | **SE (d)** | **0.43** | |
| **9** | **CI (d)** | 0.72 | |
| **10** | **Test of Interaction** | -0.29 (***P* value=0.39**) | |
|  | | | |
| **11** | **RRR (=exp(d) )** | 0.88 | |
| **12** | **CI (RRR)** | 0.38-2.05 | |

| **Supplemental Table 6.** Clinical factors and 30-day mortality stratified by sex and treatment strategy in **older patients (age ≥ 65 years old) presenting with NSTEMI:** inverse probability of treatment weighting | | | | | | |
| --- | --- | --- | --- | --- | --- | --- |
|  | **Women ≥ 65 years old** | | | **Men ≥ 65 years old** | | |
|  | **Early invasive strategy**  **N=472** | **Initial conservative treatment**  **N=694** | **Standardized mean difference** | **Early invasive strategy**  **N=875** | **Initial conservative treatment**  **N=964** | **Standardized mean difference** |
| Age, years, mean (SD) | 74.8 (6.3) | 74.9 (6.8) | -0.02 | 73.3 (5.6) | 73.4 (6.3) | -0.0117 |
| Diabetes, % | 43.4 | 43.8 | -0.01 | 34.9 | 34.9 | 0.0009 |
| **Cardiovascular risk factors** | | | |  |  |  |
| Hypercholesterolemia, % | 47.6 | 47.5 | 0.002 | 43.9 | 43.3 | 0.0130 |
| Hypertension, % | 88.7 | 88.7 | -0.001 | 83.1 | 83.1 | -0.001 |
| Current smoking, % | 13.5 | 13.6 | -0.002 | 24.5 | 24.4 | 0.002 |
| Family history of CAD, % | 30.8 | 30.7 | 0.003 | 26.8 | 27.1 | -0.006 |
| **History of ischemic heart disease** | |  |  |  |  |  |
| Chronic coronary syndrome, % | 27.5 | 28.2 | -0.01 | 24.6 | 25.0 | -0.01 |
| Prior myocardial infarction, % | 21.8 | 21.2 | 0.01 | 25.4 | 25.6 | -0.01 |
| Prior CABG, % | 4.7 | 4.0 | 0.04 | 6.3 | 6.4 | -0.003 |
| Prior PCI, % | 11.4 | 11.3 | 0.002 | 15.7 | 15.7 | -0.002 |
| **History of cardiovascular disease** | |  |  |  |  |  |
| Peripheral artery disease, % | 4.4 | 4.4 | -0.003 | 5.3 | 5.4 | -0.002 |
| Prior heart failure, % | 8.9 | 9.0 | -0.005 | 7.0 | 7.1 | -0.005 |
| Prior stroke or TIA, % | 7.2 | 6.9 | 0.01 | 7.1 | 6.6 | 0.02 |
| **Other comorbidities** |  |  |  |  |  |  |
| Chronic kidney disease, % | 14.6 | 14.4 | 0.004 | 12.5 | 13.1 | -0.02 |
| **Clinical presentation on hospital admission** | |  |  |  |  |  |
| Heart rate, bpm, mean (SD) | 85.0 (21.5) | 85.0 (22.3) | 0.001 | 82.6 (20.8) | 82.7 (20.9) | -0.01 |
| SBP, mmHg, mean (SD) | 143.0 (25.9) | 143.2 (27.1) | -0.01 | 142.9 (25.5) | 142.7 (25.1) | 0.01 |
| **Outcomes** |  |  | ***P* value** |  |  | ***P* value** |
| 30-day mortality, % | 3.5 | 6.5 | 0.02 | 2.9 | 6.6 | 0.0002 |
| Risk ratio (95% CI) | 0.52 (0.29 – 0.93) | | 0.03 | 0.43 (0.27 – 0.69) | | 0.0004 |
| Data are weighted mean ± standard deviation or weighted rate, unless otherwise specified.  **Abbreviations:** CABG=coronary artery bypass graft; CAD=coronary artery disease; HR=heart rate; NSTEMI=non-ST-segment elevation yocardial infarction; PCI=percutaneous coronary intervention; SBP=systolic blood pressure; TIA=transient ischemic attack | | | | | | |

| **Supplemental Table 7.** Interaction test for comparing two estimated risk ratios (women vs. men) in **older patients (age ≥65 years** **old) presenting with NSTEMI**: 30-day mortality for early invasive vs. initial conservative treatment | | | |
| --- | --- | --- | --- |
|  |  | **Group 1**  **[Women ≥65 years old]**  **(n=1166)** | **Group 2**  **[Men ≥65 years old]**  **(n=1839)** |
| **1** | **RR** | 0.52 | 0.43 |
| **2** | **log RR** | -0.65 | -0.84 |
| **3** | **95% CI for RR** | (0.29 – 0.93) | (0.27 – 0.69) |
| **4** | **95% CI for log RR** | -1.23-(-0.07) | -1.31-(-0.37) |
| **5** | **Width of CI** | 1.16 | 0.94 |
| **6** | **SE (=width / (2*1.96))** | 0.30 | 0.24 |
|  | | | |
| **7** | **d (=**$\boldsymbol{E}_{\boldsymbol{1}}\boldsymbol{-}\boldsymbol{E}_{\boldsymbol{2}}$**)** | **0.19** | |
| **8** | **SE (d)** | **0.38** | |
| **9** | **CI (d)** | -0.56-0.94 | |
| **10** | **Test of Interaction** | 0.50(***P* value=0.31**) | |
|  | | | |
| **11** | **RRR (=exp(d) )** | 1.21 | |
| **12** | **CI (RRR)** | 0.57-2.56 | |

| **Supplemental Table 8.** Clinical factors and 30-day mortality stratified by sex and treatment strategy in **younger patients** (**age<65 years old) presenting with NSTEMI:** inverse probability of treatment weighting | | | | | | |
| --- | --- | --- | --- | --- | --- | --- |
|  | **Women <65 years old** | | | **Men <65 years old** | | |
|  | **Early invasive strategy**  **N=354** | **Initial conservative treatment**  **N=378** | **Standardized mean difference** | **Early invasive strategy**  **N=1175** | **Initial conservative treatment**  **N=1015** | **Standardized mean difference** |
| Age, years, mean (SD) | 56.0 (6.7) | 56.0 (6.6) | -0.01 | 54.4 (7.1) | 54.4 (7.5) | 0.001 |
| Diabetes, % | 28.7 | 28.6 | 0.004 | 22.1 | 22.2 | -0.001 |
| **Cardiovascular risk factors** | | | |  |  |  |
| Hypercholesterolemia, % | 47.0 | 46.8 | 0.004 | 44.3 | 44.4 | -0.001 |
| Hypertension, % | 75.0 | 75.5 | -0.01 | 67.3 | 67.1 | 0.003 |
| Current smoking, % | 41.0 | 41.1 | -0.002 | 52.7 | 52.5 | 0.005 |
| Family history of CAD, % | 36.2 | 36.3 | -0.003 | 35.0 | 35.3 | -0.01 |
| **History of ischemic heart disease** | |  |  |  |  |  |
| Chronic coronary syndrome, % | 19.3 | 19.2 | 0.001 | 18.4 | 18.4 | 0.0004 |
| Prior myocardial infarction, % | 17.9 | 18.4 | -0.01 | 20.0 | 20.1 | -0.002 |
| Prior CABG, % | 1.3 | 1.3 | 0.0002 | 2.4 | 2.5 | -0.01 |
| Prior PCI, % | 9.7 | 9.8 | -0.001 | 14.4 | 14.5 | -0.003 |
| **History of cardiovascular disease** | |  |  |  |  |  |
| Peripheral artery disease, % | 2.8 | 2.5 | 0.02 | 1.7 | 1.7 | -0.01 |
| Prior heart failure, % | 3.8 | 4.1 | -0.01 | 3.1 | 3.1 | 0.001 |
| Prior stroke or TIA, % | 4.1 | 4.2 | -0.01 | 3.3 | 3.3 | 0.0003 |
| **Other comorbidities** |  |  |  |  |  |  |
| Chronic kidney disease, % | 4.0 | 4.3 | -0.01 | 4.0 | 4.0 | -0.001 |
| **Clinical presentation on hospital admission** | |  |  |  |  |  |
| Heart rate, bpm, mean (SD) | 82.8 (18.8) | 82.8 (18.6) | -0.002 | 82.2 (19.4) | 82.2 (19.3) | 0.0001 |
| SBP, mmHg, mean (SD) | 146.9 (27.0) | 146.8 (25.8) | 0.004 | 144.7 (26.1) | 144.7 (24.8) | 0.004 |
| **Outcomes** |  |  | ***P* value** |  |  | ***P* value** |
| 30-day mortality, % | 2.5 | 1.2 | 0.22 | 0.9 | 2.0 | 0.04 |
| Risk ratio (95% CI) | 2.03 (0.65 – 6.31) | | 0.22 | 0.46 (0.22 – 0.97) | | 0.04 |
| Data are weighted mean ± standard deviation or weighted rate, unless otherwise specified.  **Abbreviations:** CABG=coronary artery bypass graft; CAD=coronary artery disease; HR=heart rate; NSTEMI=non-ST-segment elevation myocardial infarction; PCI=percutaneous coronary intervention; SBP=systolic blood pressure; TIA=transient ischemic attack. | | | | | | |

| **Supplemental Table 9**. Interaction test for comparing two estimated risk ratios (women vs. men) in **younger patients** (**age<65 years old) presenting with NSTEMI:**  30-day mortality for early invasive vs. initial conservative treatment | | | |
| --- | --- | --- | --- |
|  |  | **Group 1**  **[Women<65 years old]**  **(n=732)** | **Group 2**  **[Men<65 years old]**  **(n=2190)** |
| **1** | **RR** | 2.03 | 0.46 |
| **2** | **log RR** | 0.71 | -0.78 |
| **3** | **95% CI for RR** | (0.65 – 6.31) | (0.22 – 0.97) |
| **4** | **95% CI for log RR** | -0.43-(1.84) | -1.51-(-0.03) |
| **5** | **Width of CI** | 2.27 | 1.48 |
| **6** | **SE (=width / (2*1.96))** | 0.58 | 0.38 |
|  | | | |
| **7** | **d (=**$\boldsymbol{E}_{\boldsymbol{1}}\boldsymbol{-}\boldsymbol{E}_{\boldsymbol{2}}$**)** | **1.48** | |
| **8** | **SE (d)** | **0.69** | |
| **9** | **CI (d)** | 0.13-2.84 | |
| **10** | **Test of Interaction** | 2.14 (***P* value= 0.02**) | |
|  | | | |
| **11** | **RRR (=exp(d) )** | 4.41 | |
| **12** | **CI (RRR)** | 1.14-17.15 | |

| **Supplemental Table 10.** Clinical factors and 30-day mortality stratified by sex and treatment strategy in **older patients** **(age≥65 years old)** with **GRACE score >140:** inverse probability of treatment weighting | | | | | | |
| --- | --- | --- | --- | --- | --- | --- |
|  | **Women ≥65 years old** | | | **Men ≥65 years old** | | |
|  | **Early invasive strategy**  **N=256** | **Initial conservative treatment**  **N=505** | **Standardized mean difference** | **Early invasive strategy**  **N=465** | **Initial conservative treatment**  **N=660** | **Standardized mean difference** |
| Age, years, mean (SD) | 77.2 (6.3) | 77.3 (6.7) | -0.02 | 76.0 (5.9) | 76.1 (6.2) | -0.02 |
| Diabetes, % | 43.0 | 43.8 | -0.02 | 36.5 | 35.3 | 0.02 |
| **Cardiovascular risk factors** | | | |  |  |  |
| Hypercholesterolemia, % | 50.0 | 50.2 | -0.004 | 46.6 | 46.0 | 0.01 |
| Hypertension, % | 88.0 | 88.9 | -0.03 | 83.2 | 83.3 | -0.004 |
| Current smoking, % | 11.9 | 12.2 | -0.01 | 18.6 | 18.4 | 0.004 |
| Family history of CAD, % | 35.4 | 35.0 | 0.01 | 30.5 | 30.4 | 0.002 |
| **History of ischemic heart disease** | |  |  |  |  |  |
| Chronic coronary syndrome, % | 39.8 | 40.7 | -0.02 | 36.8 | 35.9 | 0.02 |
| Prior myocardial infarction, % | 23.2 | 22.6 | 0.02 | 26.5 | 28.0 | -0.04 |
| Prior CABG, % | 5.3 | 4.2 | 0.05 | 8.0 | 8.2 | -0.01 |
| Prior PCI, % | 14.8 | 14.8 | -0.001 | 17.8 | 17.9 | -0.002 |
| **History of cardiovascular disease** | |  |  |  |  |  |
| Peripheral artery disease, % | 5.7 | 5.2 | 0.02 | 6.7 | 7.4 | -0.03 |
| Prior heart failure, % | 12.8 | 13.0 | -0.004 | 10.8 | 10.9 | -0.01 |
| Prior stroke or TIA, % | 8.5 | 7.6 | 0.04 | 9.0 | 8.4 | 0.02 |
| **Other comorbidities** |  |  |  |  |  |  |
| Chronic kidney disease, % | 20.2 | 19.6 | 0.02 | 17.7 | 18.9 | -0.03 |
| **Clinical presentation on hospital admission** | |  |  |  |  |  |
| Heart rate, bpm, mean (SD) | 91.9 (28.3) | 90.2 (24.6) | 0.06 | 88.8 (27.4) | 88.3 (22.6) | 0.02 |
| SBP, mmHg, mean (SD) | 133.1 (28.2) | 134.6 (28.1) | -0.05 | 134.3 (25.9) | 134.2 (25.8) | 0.003 |
| **Outcomes** |  |  | ***P* value** |  |  | ***P* value** |
| 30-day mortality, % | 5.1 | 8.6 | 0.06 | 4.0 | 8.5 | 0.002 |
| Risk ratio (95% CI) | 0.57 (0.30 – 1.07) | | 0.08 | 0.46 (0.27 – 0.78) | | 0.004 |
| Data are weighted mean ± standard deviation or weighted rate, unless otherwise specified.  **Abbreviations**: CABG=coronary artery bypass graft; CAD=coronary artery disease; HR=heart rate; PCI=percutaneous coronary intervention; SBP=systolic blood pressure; TIA=transient ischemic attack | | | | | | |

| **Supplemental Table 11**. Interaction test for comparing two estimated risk ratios (women vs. men) in **older patients (age≥65 years old) with GRACE score >140**: 30-day for early invasive vs. initial conservative treatment | | | |
| --- | --- | --- | --- |
|  |  | **Group 1**  **[Women ≥65 years old]**  **(n=761)** | **Group 2**  **[Men ≥65 years old]**  **(n=1125)** |
| **1** | **RR** | 0.57 | 0.46 |
| **2** | **log RR** | -0.56 | -0.78 |
| **3** | **95% CI for RR** | (0.30 – 1.07) | (0.27 – 0.78) |
| **4** | **95% CI for log RR** | -1.20-0.07 | -1.31-(-0.25) |
| **5** | **Width of CI** | 1.27 | 1.06 |
| **6** | **SE (=width / (2*1.96))** | 0.32 | 0.27 |
|  | | | |
| **7** | **d (=**$\boldsymbol{E}_{\boldsymbol{1}}\boldsymbol{-}\boldsymbol{E}_{\boldsymbol{2}}$**)** | **0.21** | |
| **8** | **SE (d)** | **0.42** | |
| **9** | **CI (d)** | -0.61-1.04 | |
| **10** | **Test of Interaction** | 0.51(***P=*value 0.31**) | |
|  | | | |
| **11** | **RRR (=exp(d) )** | 1.24 | |
| **12** | **CI (RRR)** | 0.54-2.83 | |

| **Supplemental Table 12.** Clinical factors and 30-day mortality stratified by sex and treatment strategy in **younger patients** (**age<65 years old)** **with GRACE score >140**: inverse probability of treatment weighting | | | | | | |
| --- | --- | --- | --- | --- | --- | --- |
|  | **Women** <**65 years old** | | | **Men** <**65 years old** | | |
|  | **Early invasive strategy**  **N=29** | **Initial conservative treatment**  **N=52** | **Standardized mean difference** | **Early invasive strategy**  **N=112** | **Initial conservative treatment**  **N=118** | **Standardized mean difference** |
| Age, years, mean (SD) | 59.8 (4.1) | 59.7 (4.1) | 0.02 | 59.3 (4.1) | 59.2 (4.5) | 0.02 |
| Diabetes, % | 38.5 | 39.2 | -0.01 | 37.4 | 36.8 | 0.01 |
| **Cardiovascular risk factors** | | | |  |  |  |
| Hypercholesterolemia, % | 54.0 | 62.6 | -0.17 | 53.4 | 52.2 | 0.02 |
| Hypertension, % | 75.6 | 79.7 | -0.09 | 74.3 | 74.1 | 0.004 |
| Current smoking, % | 29.3 | 28.0 | 0.03 | 40.1 | 41.2 | -0.02 |
| Family history of CAD, % | 51.7 | 61.9 | -0.21 | 47.6 | 46.8 | 0.02 |
| **History of ischemic heart disease** | |  |  |  |  |  |
| Chronic coronary syndrome, n (%) | 38.6 | 42.7 | -0.08 | 41.3 | 39.8 | 0.03 |
| Prior myocardial infarction, % | 11.3 | 12.7 | -0.06 | 27.7 | 26.8 | 0.02 |
| Prior CABG, % | 6.6 | 5.2 | 0.06 | 9.8 | 8.8 | 0.04 |
| Prior PCI, % | 5.0 | 7.3 | -0.06 | 27.4 | 26.1 | 0.03 |
| **History of cardiovascular disease** | |  |  |  |  |  |
| Peripheral artery disease, % | 3.6 | 2.5 | 0.06 | 5.0 | 4.8 | 0.01 |
| Prior heart failure, % | 10.5 | 6.5 | 0.05 | 9.3 | 9.1 | 0.01 |
| Prior stroke or TIA, % | 2.1 | 3.2 | -0.07 | 2.0 | 1.8 | 0.02 |
| **Other comorbidities** |  |  |  |  |  |  |
| Chronic kidney disease, % | 9.4 | 11.1 | -0.05 | 17.2 | 16.0 | 0.03 |
| **Clinical presentation on hospital admission** | |  |  |  |  |  |
| Heart rate, bpm, mean (SD) | 89.1 (21.9) | 90.9 (25.7) | -0.05 | 98.4 (33.3) | 98.0 (28.4) | 0.01 |
| SBP, mmHg, mean (SD) | 127.9 (31.6) | 124.9 (24.8) | 0.05 | 123.1 (31.4) | 122.5 (25.2) | 0.02 |
| **Outcomes** |  |  | ***P* value** |  |  | ***P* value** |
| 30-day mortality, % | 5.3 | 0.0 | 0.22 | 4.0 | 4.1 | 0.95 |
| Risk ratio (95% CI) | - | | - | 0.96 (0.26 – 3.57) | | 0.95 |
| Data are weighted mean ± standard deviation or weighted rate, unless otherwise specified.  **Abbreviations:** CABG=coronary artery bypass graft; CAD=coronary artery disease; HR=heart rate; PCI=percutaneous coronary intervention; SBP=systolic blood pressure; TIA=transient ischemic attack | | | | | | |

| **Supplemental Table 13.** Clinical factors and 30-day mortality stratified by sex and treatment strategy in with **GRACE score<140:** inverse probability of treatment weighting | | | | | | |
| --- | --- | --- | --- | --- | --- | --- |
|  | **Women** | | | **Men** | | |
|  | **Early invasive strategy**  **N=710** | **Initial conservative treatment**  **N=897** | **Standardized mean difference** | **Early invasive strategy**  **N=1941** | **Initial conservative treatment**  **N=1843** | **Standardized mean difference** |
| Age, years, mean (SD) | 62.9±9.8 | 63.0±10.5 | -0.01 | 59.4±10.0 | 59.4±10.4 | 0.0004 |
| Diabetes, % | 33.7 | 33.5 | 0.004 | 25.1 | 25.2 | -0.004 |
| **Cardiovascular risk factors** | | | |  |  |  |
| Hypercholesterolemia, % | 50.7 | 50.8 | -0.002 | 46.8 | 46.8 | 0.0000 |
| Hypertension, % | 80.3 | 80.5 | -0.005 | 73.3 | 73.2 | 0.003 |
| Current smoking, % | 27.9 | 27.9 | 0.001 | 43.1 | 43.0 | 0.002 |
| Family history of CAD, % | 35.0 | 35.3 | -0.006 | 33.7 | 33.9 | -0.004 |
| **History of ischemic heart disease** | |  |  |  |  |  |
| Chronic coronary syndrome, % | 27.5 | 27.9 | -0.01 | 26.3 | 26.5 | -0.01 |
| Prior myocardial infarction, % | 18.7 | 19.5 | -0.02 | 22.4 | 22.5 | -0.003 |
| Prior CABG, % | 2.3 | 2.5 | -0.01 | 3.6 | 3.7 | -0.003 |
| Prior PCI, % | 11.6 | 11.6 | 0.0003 | 16.1 | 16.1 | -0.001 |
| **History of cardiovascular disease** | |  |  |  |  |  |
| Peripheral artery disease, % | 2.3 | 2.3 | -0.004 | 2.5 | 2.4 | 0.01 |
| Prior heart failure, % | 4.2 | 4.3 | -0.004 | 3.7 | 3.8 | -0.002 |
| Prior stroke or TIA, % | 4.8 | 4.9 | -0.01 | 3.6 | 3.5 | 0.01 |
| **Other comorbidities** |  |  |  |  |  |  |
| Chronic kidney disease, % | 5.1 | 5.1 | 0.0003 | 4.9 | 4.8 | 0.01 |
| **Clinical presentation on hospital admission** | |  |  |  |  |  |
| Heart rate, bpm, mean (SD) | 79.6±16.4 | 79.6±15.9 | -0.0004 | 78.8±16.2 | 78.8±16.9 | -0.001 |
| SBP, mmHg, mean (SD) | 149.2±24.3 | 149.3±23.8 | -0.002 | 146.7±24.1 | 146.6±23.8 | 0.001 |
| **Outcomes** |  |  | ***P* value** |  |  | ***P* value** |
| 30-day mortality, % | 1.4 | 1.2 | 0.76 | 1.0 | 1.9 | 0.02 |
| Risk ratio (95% CI) | 1.15 (0.48 – 2.72) | | 0.76 | 0.51 (0.29 – 0.88) | | 0.02 |
| Data are weighted mean ± standard deviation or weighted rate, unless otherwise specified.  **Abbreviations**: CABG=coronary artery bypass graft; CAD=coronary artery disease; HR=heart rate; PCI=percutaneous coronary intervention; SBP=systolic blood pressure; TIA=transient ischemic attack | | | | | | |

| **Supplemental Table 14**. Interaction test for comparing two estimated risk ratios (women vs. men) in **patients with GRACE score <140**: 30-day for early invasive vs. initial conservative treatment | | | |
| --- | --- | --- | --- |
|  |  | **Group 1**  **[Women]**  **(n=1607)** | **Group 2**  **[Men]**  **(n=3784)** |
| **1** | **RR** | 1.15 | 0.51 |
| **2** | **log RR** | 0.1398 | -0.6733 |
| **3** | **95% CI for RR** | 0.48 – 2.72 | 0.29 |
| **4** | **95% CI for log RR** | -0.7340 – 1.0006 | -1.2379 - -0.1278 |
| **5** | **Width of CI** | 1.7346 | 1.1101 |
| **6** | **SE (=width / (2*1.96))** | 0.4425 | 0.2832 |
|  | | | |
| **7** | **d (=**$\boldsymbol{E}_{\boldsymbol{1}}\boldsymbol{-}\boldsymbol{E}_{\boldsymbol{2}}$**)** | **0.8131** | |
| **8** | **SE (d)** | **0.5254** | |
| **9** | **CI (d)** | -0.2167 – 1.8429 | |
| **10** | **Test of Interaction** | 1.5476 (p-value: 0.06) | |
|  | | | |
| **11** | **RRR (=exp(d) )** | 2.2549 | |
| **12** | **CI (RRR)** | 0.8052 – 6.3148 | |

| **Supplemental Table 15**. Clinical factors and PCI related complications stratified by sex in **patients undergoing PCI**: inverse probability of treatment weighting | | | |
| --- | --- | --- | --- |
|  | **Women**  **N=1323** | **Men**  **N=3366** | **Standardized mean difference** |
| Age, years, mean (SD) | 62.9 (11.2) | 63.1 (11.0) | -0.01 |
| Diabetes, % | 28.4 | 28.7 | -0.01 |
| **Cardiovascular risk factors** | | | |
| Hypercholesterolemia, % | 48.7 | 49.4 | -0.01 |
| Hypertension, % | 76.3 | 76.5 | -0.004 |
| Current smoking, % | 37.6 | 37.4 | 0.01 |
| Family history of CAD, % | 34.2 | 34.3 | -0.004 |
| **History of ischemic heart disease** | |  |  |
| Chronic coronary syndrome, % | 27.6 | 27.5 | 0.002 |
| Prior myocardial infarction, % | 21.5 | 21.5 | 0.002 |
| Prior CABG, % | 3.1 | 3.5 | -0.02 |
| Prior PCI, % | 17.1 | 16.8 | 0.01 |
| **History of cardiovascular disease** | |  |  |
| Peripheral artery disease, % | 3.8 | 3.7 | 0.004 |
| Prior heart failure, % | 4.6 | 4.8 | -0.01 |
| Prior stroke or TIA, % | 4.0 | 4.1 | -0.01 |
| **Other comorbidities** |  |  |  |
| Chronic kidney disease, % | 7.9 | 7.9 | 0.0003 |
| **Clinical presentation on hospital admission** | |  |  |
| Heart rate, bpm, mean (SD) | 79.7 (17.6) | 80.0 (19.0) | -0.02 |
| SBP, mmHg, mean (SD) | 144.9 (25.8) | 144.8 (25.2) | 0.01 |
| **Outcomes** |  |  | ***P* value** |
| PCI related complications, % | 3.8 | 4.1 | 0.69 |
| Risk ratio (95% CI) | 0.94 (0.67 – 1.30) | | 0.69 |
| Data are weighted mean ± standard deviation or weighted rate, unless otherwise specified.  **Abbreviations:** bpm=beats per minute; CABG=coronary artery bypass graft; CAD= coronary artery disease; PCI=percutaneous coronary intervention; SBP=systolic blood pressure; TIA= Transient ischemic attack. | | | |

| **Supplemental Table 16.** Clinical factors and PCI related complications stratified by sex and treatment strategy in patients **undergoing PCI**: inverse probability of treatment weighting | | | | | | |
| --- | --- | --- | --- | --- | --- | --- |
|  | **Women** | | | **Men** | | |
|  | **Early invasive strategy**  **N=982** | **Initial conservative treatment**  **N=341** | **Standardized mean difference** | **Early invasive strategy**  **N=2477** | **Initial conservative treatment**  **N=889** | **Standardized mean difference** |
| Age, years, mean (SD) | 66.0 (10.6) | 66.3 (11.3) | -0.03 | 61.9 (10.9) | 61.9 (11.4) | 0.003 |
| Diabetes, % | 35.6 | 36.9 | -0.03 | 26.1 | 26.0 | 0.001 |
| **Cardiovascular risk factors** | | | |  |  |  |
| Hypercholesterolemia, % | 52.5 | 52.5 | -0.0004 | 48.2 | 48.3 | -0.003 |
| Hypertension, % | 82.7 | 82.9 | -0.01 | 74.1 | 74.0 | 0.002 |
| Current smoking, % | 27.7 | 27.5 | 0.003 | 41.2 | 41.0 | 0.004 |
| Family history of CAD, % | 33.9 | 35.1 | -0.02 | 34.5 | 33.9 | 0.01 |
| **History of ischemic heart disease** | |  |  |  |  |  |
| Chronic coronary syndrome, % | 28.9 | 28.1 | 0.02 | 26.8 | 26.4 | 0.01 |
| Prior myocardial infarction, % | 18.3 | 19.0 | -0.02 | 22.7 | 22.7 | 0.002 |
| Prior CABG, % | 2.9 | 3.3 | -0.03 | 3.8 | 3.7 | 0.01 |
| Prior PCI, % | 14.1 | 15.3 | -0.03 | 18.0 | 18.1 | -0.004 |
| **History of cardiovascular disease** | |  |  |  |  |  |
| Peripheral artery disease, % | 3.9 | 3.8 | 0.01 | 3.6 | 3.5 | 0.01 |
| Prior heart failure, % | 6.8 | 6.8 | 0.001 | 4.2 | 4.3 | -0.01 |
| Prior stroke or TIA, % | 5.1 | 5.2 | -0.004 | 3.9 | 3.8 | 0.004 |
| **Other comorbidities** |  |  |  |  |  |  |
| Chronic kidney disease, % | 9.7 | 10.3 | -0.02 | 7.3 | 7.5 | -0.01 |
| **Clinical presentation on hospital admission** | |  |  |  |  |  |
| Heart rate, bpm, mean (SD) | 81.1 (18.4) | 80.9 (17.9) | 0.01 | 79.5 (18.3) | 79.4 (19.5) | 0.01 |
| SBP, mmHg, mean (SD) | 146.2 (25.9) | 146.4 (24.2) | -0.01 | 144.1 (25.6) | 143.6 (23.7) | 0.02 |
| **Outcomes** |  |  | ***P* value** |  |  | ***P* value** |
| PCI related complications, % | 3.3 | 4.5 | 0.37 | 4.0 | 3.7 | 0.64 |
| Risk ratio (95% CI) | 0.74 (0.40 – 1.37) | | 0.34 | 1.10 (0.73 – 1.64) | | 0.65 |
| Data are weighted mean ± standard deviation or weighted rate, unless otherwise specified.  **Abbreviations:** CABG=coronary artery bypass graft; CAD=coronary artery disease; HR=heart rate; PCI=percutaneous coronary intervention; SBP=systolic blood pressure; TIA=transient ischemic attack | | | | | | |

| **Supplemental Table 17.** Interaction test for comparing two estimated risk ratios (women vs. men) **in patients undergoing PCI:** PCI related complications for early invasive strategy vs. initial conservative treatment | | | |
| --- | --- | --- | --- |
|  |  | **Group 1**  **[Women]**  **(n=1323)** | **Group 2**  **[Men]**  **(n=3366)** |
| **1** | **RR** | 0.74 | 1.10 |
| **2** | **log RR** | -0.30 | 0.10 |
| **3** | **95% CI for RR** | (0.40 – 1.37) | (0.73 – 1.64) |
| **4** | **95% CI for log RR** | -0.92-0.31 | -0.31-0.50 |
| **5** | **Width of CI** | 1.23 | 0.81 |
| **6** | **SE (=width / (2*1.96))** | 0.31 | 0.21 |
|  | | | |
| **7** | **d (=**$\boldsymbol{E}_{\boldsymbol{1}}\boldsymbol{-}\boldsymbol{E}_{\boldsymbol{2}}$**)** | **-0.40** | |
| **8** | **SE (d)** | **0.38** | |
| **9** | **CI (d)** | -1.13-0.34 | |
| **10** | **Test of Interaction** | -1.05 (***P* value=0.15**) | |
|  | | | |
| **11** | **RRR (=exp(d) )** | 0.67 | |
| **12** | **CI (RRR)** | 0.32-1.41 | |

| **Supplemental Table 18.** Clinical factors and **major bleeding complications** stratified by sex in **the overall population:** inverse probability of treatment weighting | | | |
| --- | --- | --- | --- |
|  | **Women**  **N=2450** | **Men**  **N=5139** | **Standardized mean difference** |
| Age, years, mean (SD) | 64.2 (11.8) | 64.4 (11.5) | -0.01 |
| Diabetes, % | 30.8 | 30.9 | -0.003 |
| **Cardiovascular risk factors** | | | |
| Hypercholesterolemia, % | 47.9 | 48.4 | -0.01 |
| Hypertension, % | 77.6 | 77.7 | -0.002 |
| Current smoking, % | 33.2 | 32.8 | 0.01 |
| Family history of CAD, % | 34.6 | 34.9 | -0.01 |
| **History of ischemic heart disease** | |  |  |
| Chronic coronary syndrome, % | 30.0 | 30.2 | -0.005 |
| Prior myocardial infarction, % | 23.0 | 23.2 | -0.004 |
| Prior CABG, % | 4.0 | 4.3 | -0.01 |
| Prior PCI, % | 15.7 | 15.6 | 0.003 |
| **History of cardiovascular disease** | |  |  |
| Peripheral artery disease, % | 3.8 | 3.7 | 0.005 |
| Prior heart failure, % | 5.9 | 6.0 | -0.01 |
| Prior stroke or TIA, % | 4.9 | 4.9 | 0.0003 |
| **Other comorbidities** |  |  |  |
| Chronic kidney disease, % | 8.8 | 8.9 | -0.004 |
| **Clinical presentation on hospital admission** | |  |  |
| Heart rate, bpm, mean (SD) | 82.0 (19.7) | 82.2 (20.1) | -0.01 |
| SBP, mmHg, mean (SD) | 143.4 (26.2) | 143.3 (25.8) | 0.001 |
| **Outcomes** |  |  | ***P* value** |
| Major bleeding, % | 1.6 | 1.5 | 0.69 |
| Risk ratio (95% CI) | 1.08 (0.73 – 1.59) | | 0.69 |
| Data are weighted mean ± standard deviation or weighted rate, unless otherwise specified.  **Abbreviations:** CABG=coronary artery bypass graft; CAD=coronary artery disease; HR=heart rate; PCI=percutaneous coronary intervention; SBP=systolic blood pressure; TIA=transient ischemic attack | | | |

| **Supplemental Table 19.** Clinical factors and **major bleeding complications** stratified by sex and treatment strategy in **the overall population:** inverse probability of treatment weighting | | | | | | |
| --- | --- | --- | --- | --- | --- | --- |
|  | **Women** | | | **Men** | | |
|  | **Early invasive strategy**  **N=995** | **Initial conservative treatment**  **N=1455** | **Standardized mean difference** | **Early invasive strategy**  **N=2518** | **Initial conservative treatment**  **N=2621** | **Standardized mean difference** |
| Age, years, mean (SD) | 67.2 (10.6) | 67.3 (11.8) | -0.01 | 62.9 (10.9) | 62.9 (12.1) | -0.004 |
| Diabetes, % | 36.5 | 36.7 | -0.01 | 27.7 | 27.8 | -0.004 |
| **Cardiovascular risk factors** | | | |  |  |  |
| Hypercholesterolemia, % | 51.0 | 51.0 | 0.001 | 47.0 | 47.0 | -0.001 |
| Hypertension, % | 82.4 | 82.8 | -0.01 | 75.2 | 75.1 | 0.002 |
| Current smoking, % | 23.0 | 23.0 | -0.002 | 37.8 | 37.7 | 0.003 |
| Family history of CAD, % | 36.4 | 36.1 | 0.01 | 34.3 | 34.2 | 0.0004 |
| **History of ischemic heart disease** | |  |  |  |  |  |
| Chronic coronary syndrome, % | 32.3 | 32.6 | -0.01 | 28.9 | 29.0 | -0.004 |
| Prior myocardial infarction, % | 20.3 | 20.6 | -0.01 | 24.1 | 24.3 | -0.005 |
| Prior CABG, % | 3.3 | 3.1 | 0.01 | 4.8 | 4.9 | -0.005 |
| Prior PCI, % | 12.6 | 12.6 | 0.001 | 17.2 | 17.1 | 0.002 |
| **History of cardiovascular disease** | |  |  |  |  |  |
| Peripheral artery disease, % | 3.4 | 3.4 | -0.003 | 3.6 | 3.6 | -0.003 |
| Prior heart failure, % | 7.2 | 7.2 | 0.003 | 5.5 | 5.5 | -0.0001 |
| Prior stroke or TIA, % | 5.9 | 5.7 | 0.01 | 4.5 | 4.6 | -0.003 |
| **Other comorbidities** |  |  |  |  |  |  |
| Chronic kidney disease, % | 10.0 | 9.8 | 0.01 | 8.3 | 8.3 | -0.001 |
| **Clinical presentation on hospital admission** | |  |  |  |  |  |
| Heart rate, bpm, mean (SD) | 83.6 (20.9) | 83.4 (20.3) | 0.01 | 81.8 (21.0) | 81.7 (19.6) | 0.01 |
| SBP, mmHg, mean (SD) | 143.7 (26.3) | 143.9 (26.4) | -0.01 | 142.8 (26.0) | 142.8 (25.2) | 0.0001 |
| **Outcomes** |  |  | ***P* value** |  |  | ***P* value** |
| Major bleeding, % | 1.1 | 1.7 | 0.24 | 0.9 | 1.8 | 0.003 |
| Risk ratio (95% CI) | 0.66 (0.33 – 1.35) | | 0.26 | 0.47 (0.28 – 0.79) | | 0.0043 |
| Data are weighted mean ± standard deviation or weighted rate, unless otherwise specified.  **Abbreviations:** CABG=coronary artery bypass graft; CAD=coronary artery disease; HR=heart rate; PCI=percutaneous coronary intervention; SBP=systolic blood pressure; TIA=transient ischemic attack | | | | | | |

| **Supplemental Table 20**. Interaction test for comparing two estimated risk ratios (women vs. men): **major bleeding complications** in the overall population for early invasive vs. initial conservative treatment | | | |
| --- | --- | --- | --- |
|  |  | **Group 1**  **[Women]**  **(n=2450)** | **Group 2**  **[Men]**  **(n=5139)** |
| **1** | **RR** | 0.66 | 0.47 |
| **2** | **log RR** | -0.42 | -0.76 |
| **3** | **95% CI for RR** | (0.33 – 1.35) | (0.28 – 0.79) |
| **4** | **95% CI for log RR** | -1.11-0.30 | -1.27-(-0.24) |
| **5** | **Width of CI** | 1.41 | 1.04 |
| **6** | **SE (=width / (2*1.96))** | 0.36 | 0.26 |
|  | | | |
| **7** | **d (=**$\boldsymbol{E}_{\boldsymbol{1}}\boldsymbol{-}\boldsymbol{E}_{\boldsymbol{2}}$**)** | **0.34** | |
| **8** | **SE (d)** | **0.45** | |
| **9** | **CI (d)** | -0.53-1.21 | |
| **10** | **Test of Interaction** | 0.76 (***P* value=0.22**) | |
|  | | | |
| **11** | **RRR (=exp(d) )** | 1.40 | |
| **12** | **CI (RRR)** | 0.59-3.37 | |

| **Supplemental Table 21.** Clinical factors and 30-day mortality stratified by age group and treatment strategy in men: inverse probability of treatment weighting **(analysis incorporating patients undergoing coronary angiography without PCI**) | | | | | | |
| --- | --- | --- | --- | --- | --- | --- |
|  | **Men age <65 years old** | | | **Men age ≥65 years old** | | |
|  | **Early invasive strategy**  **(n=1519)** | **Initial conservative strategy**  **(n=1467)** | **Standardized mean difference** | **Early invasive strategy**  **(n=1141)** | **Initial conservative strategy**  **(n=1356)** | **Standardized mean difference** |
| Age, years, mean (SD) | 54.6 (7.0) | 54.6 (7.3) | -0.001 | 73.5 (5.9) | 73.5 (6.4) | -0.01 |
| Diabetes, % | 22.6 | 22.7 | -0.002 | 34.6 | 34.4 | 0.003 |
| **Cardiovascular risk factors** | |  |  |  |  |  |
| Hypercholesterolemia, % | 48.2 | 48.3 | -0.002 | 45.7 | 45.7 | -0.002 |
| Hypertension, % | 68.8 | 68.7 | 0.001 | 84.0 | 83.8 | 0.01 |
| Current smoking, % | 50.1 | 50.0 | 0.002 | 21.7 | 21.6 | 0.002 |
| Family history of CAD, % | 38.4 | 38.6 | -0.003 | 27.7 | 27.9 | -0.004 |
| **History of ischemic heart disease** | |  |  |  |  |  |
| Chronic coronary syndrome, % | 26.4 | 26.4 | -0.001 | 31.5 | 32.0 | -0.01 |
| Prior myocardial infarction, % | 21.8 | 21.9 | -0.02 | 26.8 | 27.4 | -0.01 |
| Prior CABG, % | 3.2 | 3.4 | -0.01 | 6.6 | 6.7 | -0.004 |
| Prior PCI, % | 16.2 | 16.3 | -0.001 | 17.6 | 17.6 | -0.002 |
| **History of cardiovascular disease** | |  |  |  |  |  |
| Peripheral artery disease, % | 1.9 | 2.0 | -0.003 | 5.4 | 5.6 | -0.01 |
| Prior heart failure, % | 4.1 | 4.1 | -0.002 | 8.4 | 8.4 | 0.0004 |
| Prior stroke or TIA, % | 2.8 | 2.8 | -0.004 | 6.3 | 6.4 | -0.004 |
| **Other comorbidities** | |  |  |  |  |  |
| Chronic kidney disease, % | 4.2 | 4.3 | -0.001 | 13.1 | 13.0 | 0.002 |
| **Clinical presentation on hospital admission** | | |  |  |  |  |
| Heart rate, bpm, mean (SD) | 81.2 (18.7) | 81.2 (19.7) | -0.003 | 81.9 (21.9) | 81.9 (20.6) | 0.001 |
| SBP, mmHg, mean (SD) | 143.3 (25.8) | 143.2 (25.5) | 0.002 | 141.1 (26.3) | 141.2 (26.8) | -0.001 |
| **Outcomes** | |  | ***P* value** |  |  | ***P* value** |
| 30-day mortality, % | 0.9 | 1.9 | 0.02 | 3.9 | 5.6 | 0.04 |
| Risk ratio (95% CI) | 0.47 (0.24 – 0.89) | | 0.02 | 0.68 (0.47 – 0.99) | | 0.05 |
| Data are expressed as weighted means (standard deviation) or weighted percentages, unless otherwise specified.  **Abbreviations:** CABG=coronary artery bypass graft; CAD=coronary artery disease; HR=heart rate; PCI=percutaneous coronary intervention; SBP=systolic blood pressure; TIA=transient ischemic attack | | | | | | |

| **Supplemental Table 22**. Interaction test for comparing two estimated risk ratios in men (age<65 years old vs. age≥65 years old): 30-day mortality for early invasive strategy vs. initial conservative strategy (analysis incorporating patients undergoing coronary angiography without PCI) | | | |
| --- | --- | --- | --- |
|  |  | **Group 1**  **[Men age <65 years old]**  **(n=2986)** | **Group 2**  **[Men age ≥65 years old]**  **(n=2497)** |
| **1** | **RR** | 0.47 | 0.68 |
| **2** | **log RR** | -0.76 | -0.39 |
| **3** | **95% CI for RR** | (0.24 – 0.89) | (0.47 – 0.99) |
| **4** | **95% CI for log RR** | -1.43 (-0.12) | -0.76-(-0.01) |
| **5** | **Width of CI** | 1.31 | 0.74 |
| **6** | **SE (=width / (2*1.96))** | 0.33 | 0.19 |
|  | | | |
| **7** | **d (=**$\boldsymbol{E}_{\boldsymbol{1}}\boldsymbol{-}\boldsymbol{E}_{\boldsymbol{2}}$**)** | **-0.37** | |
| **8** | **SE (d)** | **0.38** | |
| **9** | **CI (d)** | -1.12-0.38 | |
| **10** | **Test of Interaction** | -0.96 (***P* value=0.17**) | |
|  | | | |
| **11** | **RRR (=exp(d) )** | 0.69 | |
| **12** | **CI (RRR)** | 0.33-1.47 | |

| **Supplemental Table 23.** Clinical factors and 30-day mortality stratified by age group and treatment strategy in women: inverse probability of treatment weighting **(analysis incorporating patients undergoing coronary angiography without PCI**) | | | | | | |
| --- | --- | --- | --- | --- | --- | --- |
|  | **Women age <65 years old** | | | **Women age ≥65 years old** | | |
|  | **Early invasive strategy**  **(n=443)** | **Initial conservative strategy**  **(n=563)** | **Standardized mean difference** | **Early invasive strategy**  **(n=630)** | **Initial conservative strategy**  **(n=977)** | **Standardized mean difference** |
| Age, years, mean (SD) | 55.9 (6.7) | 56.0 (6.8) | -0.01 | 74.8 (6.5) | 74.9 (6.6) | -0.01 |
| Diabetes, % | 27.6 | 27.8 | -0.01 | 42.0 | 42.0 | -0.0003 |
| **Cardiovascular risk factors** | |  |  |  |  |  |
| Hypercholesterolemia, % | 50.1 | 50.1 | -0.0004 | 49.9 | 49.7 | 0.003 |
| Hypertension, % | 74.0 | 74.7 | -0.02 | 87.9 | 88.2 | -0.01 |
| Current smoking, % | 38.5 | 38.8 | -0.01 | 12.0 | 12.0 | 0.001 |
| Family history of CAD, % | 40.8 | 40.7 | 0.004 | 32.7 | 32.6 | 0.003 |
| **History of ischemic heart disease** | |  |  |  |  |  |
| Chronic coronary syndrome, % | 27.1 | 26.9 | 0.003 | 34.7 | 35.2 | -0.01 |
| Prior myocardial infarction, % | 17.1 | 17.7 | -0.02 | 22.0 | 22.1 | -0.002 |
| Prior CABG, % | 1.4 | 1.6 | -0.02 | 4.4 | 3.9 | 0.02 |
| Prior PCI, % | 10.8 | 10.8 | -0.001 | 13.3 | 13.2 | 0.005 |
| **History of cardiovascular disease** | |  |  |  |  |  |
| Peripheral artery disease, % | 2.2 | 2.1 | 0.01 | 3.8 | 3.8 | 0.002 |
| Prior heart failure, % | 4.0 | 4.2 | -0.01 | 9.7 | 9.6 | 0.002 |
| Prior stroke or TIA, % | 3.6 | 3.7 | -0.004 | 7.2 | 7.0 | 0.01 |
| **Other comorbidities** | |  |  |  |  |  |
| Chronic kidney disease, % | 4.3 | 4.2 | 0.01 | 12.9 | 13.0 | -0.004 |
| **Clinical presentation on hospital admission** | | |  |  |  |  |
| Heart rate, bpm, mean (SD) | 82.9 (19.5) | 82.7 (20.8) | 0.01 | 83.9 (21.1) | 84.2 (21.4) | -0.01 |
| SBP, mmHg, mean (SD) | 145.5 (26.9) | 145.7 (26.2) | -0.01 | 143.3 (25.8) | 143.4 (26.9) | -0.004 |
| **Outcomes** | |  | ***P* value** |  |  | ***P* value** |
| 30-day mortality, % | 2.1 | 0.9 | 0.11 | 3.8 | 5.2 | 0.20 |
| Risk ratio (95% CI) | 2.50 (0.83 – 7.55) | | 0.11 | 0.73 (0.44 – 1.20) | | 0.21 |
| Data are expressed as weighted means (standard deviation) or weighted percentages, unless otherwise specified.  **Abbreviations:** CABG=coronary artery bypass graft; CAD=coronary artery disease; HR=heart rate; PCI=percutaneous coronary intervention; SBP=systolic blood pressure; TIA=transient ischemic attack | | | | | | |

| **Supplemental Table 24.** Interaction test for comparing two estimated risk ratios in women (age<65 years old vs. age≥65 years old): 30-day mortality for early invasive strategy vs. initial conservative strategy (analysis incorporating patients undergoing coronary angiography without PCI) | | | |
| --- | --- | --- | --- |
|  |  | **Group 1**  **[Women age <65 years old]**  **(n=1006)** | **Group 2**  **[Women age ≥65 years old]**  **(n=1607)** |
| **1** | **RR** | 2.50 | 0.73 |
| **2** | **log RR** | 0.92 | -0.31 |
| **3** | **95% CI for RR** | (0.83 – 7.55) | (0.44 – 1.20) |
| **4** | **95% CI for log RR** | -0.19-2.02 | -0.82-0.18 |
| **5** | **Width of CI** | 2.21 | 1.00 |
| **6** | **SE (=width / (2*1.96))** | 0.56 | 0.26 |
|  | | | |
| **7** | **d (=**$\boldsymbol{E}_{\boldsymbol{1}}\boldsymbol{-}\boldsymbol{E}_{\boldsymbol{2}}$**)** | **1.23** | |
| **8** | **SE (d)** | **0.62** | |
| **9** | **CI (d)** | 0.02-2.44 | |
| **10** | **Test of Interaction** | 1.99 (***P* value = 0.02**) | |
|  | | | |
| **11** | **RRR (=exp(d) )** | 3.42 | |
| **12** | **CI (RRR)** | 1.02-11.51 | |

# **REFERENCES**

1. National Health Interview Survey- Adult Tobacco Use Information. 2017. <https://www.cdc.gov/nchs/nhis/tobacco/tobacco_glossary.htm>.
2. Buuren S, Groothuis-Oudshoorn C. MICE: Multivariate Imputation by Chained Equations in R. Journal of Statistical Software 2011; 45.
3. Austin PC, Stuart EA. Moving towards best practice when using inverse probability of treatment weighting (IPTW) using the propensity score to estimate causal treatment effects in observational studies. Stat Med 2015; 34(28): 3661-79
4. Katz D, Baptista J, Azen SP, et al. Obtaining Confidence Intervals for the Risk Ratio in Cohort Studies. *Biometrics* 1978;34(3):469-74. doi: 10.2307/2530610
5. Dongsheng Y, Dalton JE. A unified approach to measuring the effect size between two groups using SAS®: SAS global forum 2012: statistics and data analysis. SAS Global Forum. 2012: 335-2012. <https://support.sas.com/resources/papers/proceedings12/335-2012.pdf>.
